# Direct Formation of Stable 1T′ Molybdenum Telluride (MoTe_2_) by Laser Annealing Processes as Robust Contacts for High-Performance Molybdenum Disulfide (MoS_2_) Field Effect Transistors

**DOI:** 10.1021/acsami.5c12868

**Published:** 2025-10-14

**Authors:** Yao-Zen Kuo, Ming-Jin Liu, Paul Albert Sino, Po-Chien Lai, Chia-Chen Chung, Yu-Chieh Hsu, Tzu-Yi Yang, Ruei-Hong Cyu, Feng-Chuan Chuang, Hao-Chung Kuo, Seokwoo Jeon, Yu-Lun Chueh

**Affiliations:** † Department of Materials Science and Engineering, 34881 National Tsing Hua University, Hsinchu 30013, Taiwan; ‡ College of Semiconductor Research, National Tsing-Hua University, Hsinchu 30013, Taiwan; § Department of Physics, 34874 National Sun Yat-Sen University, Kaohsiung 80424, Taiwan; ∥ Physics Division, National Center for Theoretical Sciences, Hsinchu 30013, Taiwan; ⊥ Center for Theoretical and Computational Physics, National Sun Yat-Sen University, Kaohsiung 80424, Taiwan; # Department of Physics, National Tsing Hua University, Hsinchu 30013, Taiwan; ∇ Department of Photonics, College of Electrical and Computer Engineering, 34914 National Yang Ming Chiao Tung University, Hsinchu 30010, Taiwan; ○ Semiconductor Research Center, Hon Hai Research Institute, Taipei 11492, Taiwan; ◆ Department of Materials Science and Engineering, 34973 Korea University, Seoul 02841, Republic of Korea

**Keywords:** 1T′-MoTe_2_, laser annealing, high-temperature stability, MoS_2_ field-effect transistor, CMOS compatibility, semimetal electrodes

## Abstract

Semimetals, with their low density of states near the Fermi level, effectively suppress metal-induced gap states, enabling near-zero Schottky barrier heights in two-dimensional (2D) field-effect transistors (FETs). However, the low melting point of semimetals (Bi, Sb) limits the applications of 2D FETs at high temperatures. Here, we introduce a robust semimetal contact strategy for high-performance MoS_2_ FETs, based on continuous-wave laser annealing of elemental metals (Bi, Mo, Pt, W) and tellurium to form metal tellurides. Specifically, the phase of molybdenum telluride (1T′/2H) can be prepared under different laser parameters. In particular, large-area, high-crystallinity films of stable 1T′-MoTe_2_ can be synthesized uniformly, and source/drain electrodes of 1T′-MoTe_2_ are formed directly from elemental Mo and Te without damaging the underlying MoS_2_. Electrical characterization shows that MoS_2_ FETs with 1T′-MoTe_2_ contacts match the performance of Bicontacted devices and maintain device uniformity over large areas. Moreover, 1T′-MoTe_2_ electrodes sustain excellent on/off ratios of ∼10^8^ after an annealing temperature at 400 °C and ∼10^5^ after 500 °C, whereas Bi contacts fail at 400 °C. This laser annealing approach offers a scalable method to fabricate robust electrodes for 2D transistors, paving the way for high-temperature CMOS applications.

## Graphical abstract

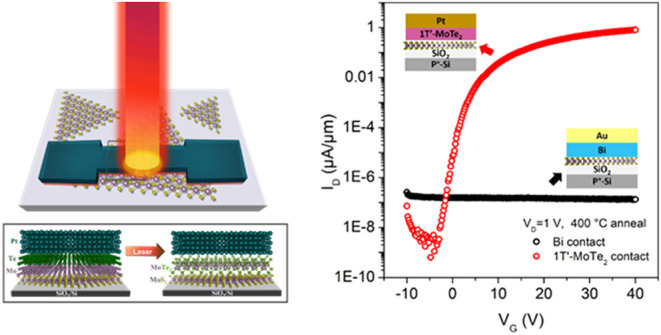

## Introduction

Monolayer transition metal dichalcogenides (TMDs), particularly molybdenum disulfide (MoS_2_), have been identified by leading semiconductor companies as promising channel materials for future advanced-node metal-oxide-semiconductor field-effect transistors (MOSFETs).^1^ Owing to its atomically thin structure and absence of dangling bonds, MoS_2_ effectively suppresses short-channel effects even in highly scaled devices^2^ while achieving carrier mobilities exceeding 100 cm^2^/V·s.^3^ However, one of the significant challenges in realizing high-performance monolayer TMDs FETs is the typically high contact resistance between the source/drain metal and the semiconductor channel.^4^ This issue primarily arises from a pronounced Fermi-level pinning (FLP) effect at the metal/TMDs interface, primarily due to the penetration of the metal wave function into the semiconductor bandgap, resulting in metal-induced gap states (MIGS)^5^ and defect-induced gap states (DIGS)^6^ caused by interface disorder or defects, together leading to elevated contact resistance.

Over the past several decades, numerous studies have focused on optimizing metal/semiconductor interface engineering to mitigate the effects of FLP. For instance, semimetal bismuth (Bi), owing to its low density of states near the Fermi level, has been demonstrated to effectively suppress MIGS, thereby significantly enhancing the performance of MoS_2_ n-type MOSFETs.^7^ Additionally, van der Waals (vdW) contact techniques have been shown to reduce interface disorder and defects, further suppressing DIGS. For example, by transferring Ag/Au, the resulting vdW metal–MoS_2_ interface achieves an atomically clean and sharply defined electronic structure, concurrently reducing tunneling currents and FLP effects.^8^ Another study reported that by lowering the substrate temperature to approximately 100 K during metal deposition and employing a lower In evaporation temperature (approximately 530 °C), a high-quality vdW In/MoS_2_ interface was fabricated, significantly reducing the contact resistance and improving device performance.^9^ Recently, Ren et al. utilized the highly conductive Dirac metal 1T-PtTe_2_ as the top-contact electrode for 2H-MoS_2_ FETs and successfully transferred it onto MoS_2_ in a nondestructive manner, thereby forming an almost ideal metal–semiconductor interface.^10^ This approach yielded an almost zero Schottky barrier and achieved extremely low contact resistance (approximately 1.58 kΩ·μm).^10^ Despite these significant advances in reducing contact resistance, these techniques still face notable limitations regarding thermal stability and large-area uniform processing. In particular, high-temperature annealing often leads to metal oxidation and interface degradation in CMOS processing,^11^ ultimately compromising the long-term stability and performance of the devices.

In recent years, two-dimensional van der Waals MoTe_2_ has attracted widespread attention as a promising contact material. It is well-known that both the 2H phase and the monoclinic 1T′-MoTe_2_ phase exhibit stability at room temperature and can be obtained as few-layer crystals via chemical vapor deposition (CVD) or mechanical exfoliation.^12^ In particular, 1T′-MoTe_2_ has become a focal point of research because of its unique physical properties, including giant magnetoresistance,^12^ pressure-induced superconductivity,^13^ type-II Weyl semimetallic behavior,^14^ and high-temperature stability.^15^ Moreover, as a two-dimensional semimetal, 1T′-MoTe_2_ has demonstrated significant potential as a contact layer in MoS_2_ FETs by effectively suppressing MIGS resulting from the decay of three-dimensional metal wave functions, thereby mitigating FLP.^16^ Furthermore, the high decomposing temperature (∼350 °C) of 1T′-MoTe_2_ is particularly advantageous in high-power or high-temperature electronic devices where contact stability under extreme conditions is crucial.^15^ Conventionally deposited metal electrodes, particularly Au, degrade after annealing at 350 °C for 1 h.^17^ Conversely, MoTe_2_-based devices have been reported to withstand temperatures over 598 K (325 °C) while maintaining their functionality and electrical performance.^18^ However, despite the promising properties of 1T′-MoTe_2_, conventional synthesis methods such as CVD and mechanical transfer are often complex, time-consuming, and struggle to achieve uniform large-area deposition, severely limiting their industrial scalability.

In this regard, we introduce a novel one-step continuous-wave (CW) laser annealing method for synthesizing 1T′-MoTe_2_. This enables the rapid formation of high-quality, uniform, large-area 1T′-MoTe_2_ contact electrodes, which retain excellent electrical performance even after high-temperature annealing, thereby satisfying the stringent thermal stability requirements of BEOL (back-end-of-line) processes. We thoroughly investigated fundamental principles and process parameters underlying the laser annealing synthesis of 1T′-MoTe_2_. We achieved controllable phase engineering between 1T′ and 2H MoTe_2_ by systematically tuning laser dwell time and utilizing quartz substrates. Raman spectroscopy, X-ray photoelectron spectroscopy (XPS), and transmission electron microscopy (TEM) results of the Mo/Te/Mo sandwich structure confirmed that the material exhibits exceptional crystallinity and uniformity. Further experiments demonstrated that this method is applicable to various transition metal combinations and can be implemented on different substrates (e.g., quartz and Si), highlighting its excellent scalability. Ultimately, by evaluating the electrical and thermal stability of the 1T′-MoTe_2_ contact layer under different annealing conditions, we demonstrated that the material maintains stable *I*_d_–*V*_g_ characteristics and high on/off ratios even under extreme thermal environments (up to 500 °C). Density functional theory (DFT) calculations were also conducted to understand the mechanism of the reduction of contact resistance in MoTe_2_ contacts. These results confirm its reliability under BEOL processes and provide an innovative, feasible technological pathway for incorporating 2D material devices into high-temperature CMOS processes.

## Results and Discussion

Figure 1a illustrates the fundamental concept of synthesizing transition metal tellurides (MTe_2_) via the CW laser annealing process with a wavelength of 808 nm. Initially, electron-beam evaporation was used to sequentially deposit a 2 nm metal layer, a 10 nm Te layer, and a 2 nm metal capping layer onto a Si/300 nm SiO_2_ substrate, forming the characteristic sandwich architecture. This architecture was designed to ensure an ample and uniform supply of reactants during the solid-state reaction and to create a stable reaction environment. The annealing process was conducted under a vacuum of approximately ∼10^–4^ Torr to prevent oxidation of the film, and the high absorption of the 808 nm laser by the Si substrate ensured uniform heating across the entire film, thereby promoting atomic diffusion and phase transformation. Ultimately, the solid-state reaction successfully synthesized MTe_2_,^19^ confirming the feasibility of the CW laser annealing process as an efficient thin-film synthesis technique. In this study, Mo was selected as the representative metal layer for further detailed investigation. Figure 1b displays Raman spectra of the annealed Mo/Te/Mo structure, revealing the characteristic A_g_ and B_g_ vibrational modes of 1T′-MoTe_2_. The peak positions remain stable under various laser power conditions, indicating that the synthesized 1T′-MoTe_2_ exhibits uniform thickness and minimal internal strain. When the metal capping layer was replaced with Al_2_O_3_, an increase in laser power induced a slight blue shift in the A_g_ mode,^20^ suggesting that CW laser with the higher energy promotes the formation of thicker 1T′-MoTe_2_ layers, as shown in Figure 1c. This effect can be attributed to the density of the capping layer material. With a Mo capping layer, Te diffuses more readily into Mo, and the reaction is primarily interface-controlled, resulting in a slight variation in product thickness. In contrast, the denser Al_2_O_3_ layer imposes diffusion limitations so that higher laser energy significantly elevates the reaction temperature, facilitating the interaction between Te and a thicker Mo layer, ultimately leading to a thicker 1T′-MoTe_2_ film. Conversely, when no capping layer was used (Te capping case), as shown in Figure 1d, the high volatility of Te results in its partial or complete evaporation prior to the onset of the solid-state reaction, thereby hindering the full conversion to MoTe_2_. These observations clearly demonstrate that the capping layer is critical for suppressing Te evaporation, maintaining a stable reactant ratio, and enhancing both the quality and uniformity of the synthesized product, as well as enabling precise control over the number of layers. To verify the universality of the process, we substituted Mo with other transition metals (e.g., Bi, Pt, and W) and subjected them to identical laser annealing conditions. The results indicate that lower-melting-point Bi requires lower laser energy (∼3 W), whereas higher-melting-point Pt and W require higher laser energy (∼8 W) to achieve effective conversion. The Raman spectra of Bi_2_Te_3_, PtTe_2_, and WTe_2_, as shown in Figure 1e,g are in good agreement with literature values,^21–23^ with narrow FWMH confirming their high crystallinity. These findings demonstrate that this method not only offers broad material diversity but also achieves excellent phase transition control, thereby underscoring its potential for scalable and high-quality synthesis of various transition metal tellurides. During our screening of potential contact-layer materials, we found that although Bi_2_Te_3_ exhibited excellent contact-resistance reduction, its low melting point rendered it unsuitable for the thermal-stability requirements of BEOL processing. PtTe_2_, despite its high electrical conductivity, is constrained by the scarcity and expense of Pt, limiting its scalability. WTe_2_, on the other hand, demands elevated e-beam evaporation temperatures because of a high melting point of W, which risks degradation of the metal/semiconductor interface. Consequently, we selected MoTe_2_ as the contact-layer material because of outstanding thermal stability with the ability to form high-quality films at controllable e-beam deposition temperatures. We subsequently performed systematic structural and electrical characterizations of the MoTe_2_ films to validate their suitability for high-temperature BEOL environments.

**Figure 1:**
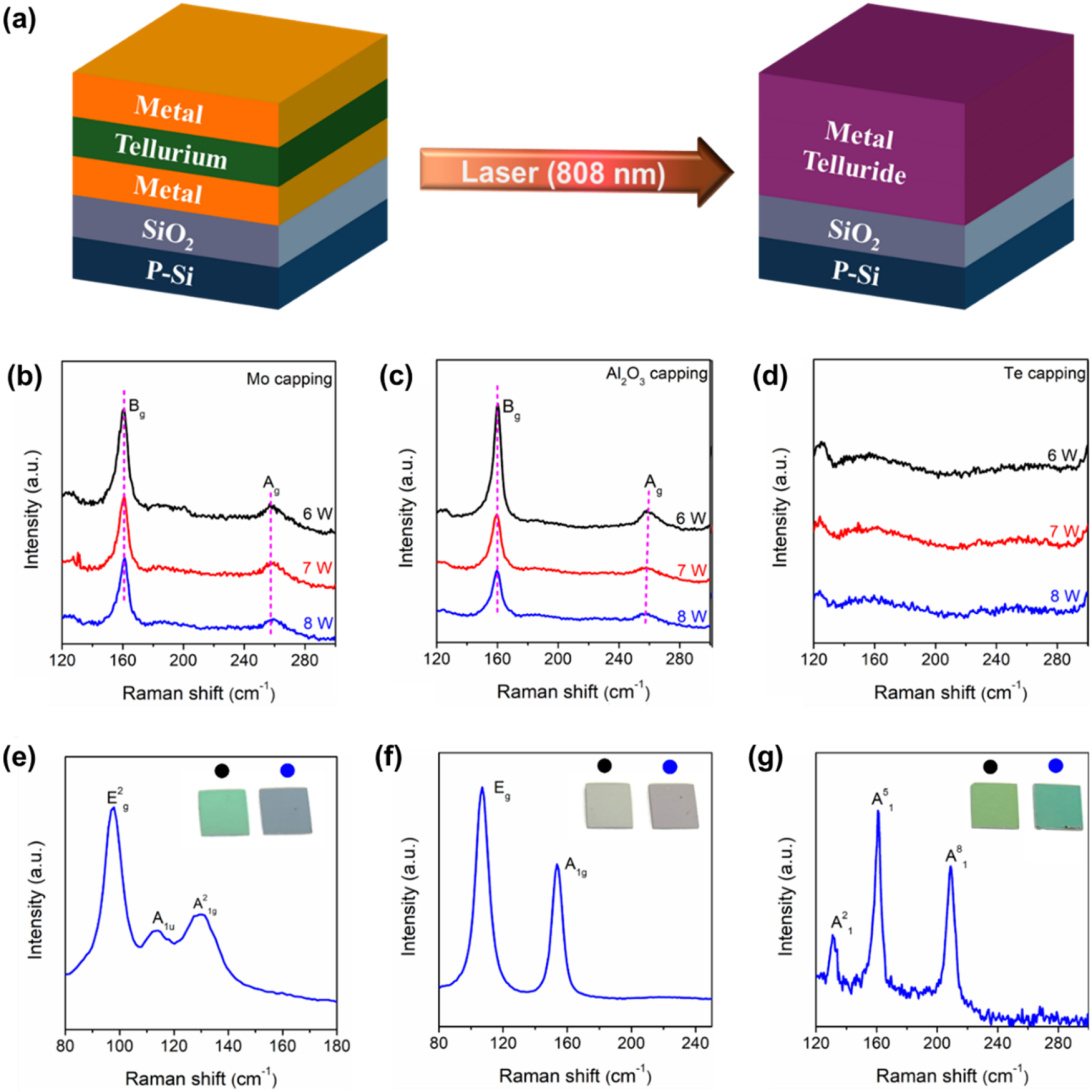
(a) Schematic figures of the laser annealing process for the formation of metal tellurides. (b–d) Overlaid Raman spectra acquired at laser powers of 6, 7, and 8 W (with an irradiation time of 1 min for all conditions), corresponding to samples with different capping layers: (b) Mo capping, (c) Al_2_O_3_ capping, and (d) Te capping. (e) Raman spectrum of Bi_2_Te_3_, (f) Raman spectrum of PtTe_2_, and (g) Raman spectrum of WTe_2_. In panels (e)–(g), black markers denote the preannealed sample, while blue markers represent the samples after the laser annealing process.

Raman spectroscopy was used to analyze the reaction stages of the Mo/Te/Mo sandwich structure under varying laser power and irradiation times. Figure 2a summarizes the effects of different laser power and exposure times on the synthesized results, with phase regions distinguished by color. Note that the sample size is 5 mm^2^. The gray region indicates insufficient laser energy, failing to induce chemical reactions effectively. The light blue region corresponds to the initial formation stage of MoTe_2_, where the broad A_g_ mode suggests low crystallinity (black curve in Figure 2b). In the optimized synthesis procedure, we obtained the 1T′-MoTe_2_ film in a green region with a larger scale (red curve in Figure 2b). However, the brick red region indicates excessive energy input, leading to the decomposition of MoTe_2_ and the formation of MoO_2_ (Blue curve in Figure 2b). Although large-area synthesis demands higher power and longer exposure times, the reaction trends remain consistent with smaller samples, demonstrating the scalability of this process (Figure S1). Previous studies have suggested that prolonged annealing of 1T′-MoTe_2_ in a Te-rich atmosphere leads to the formation of the thermodynamically stable 2H-MoTe_2_ phase.^24,25^ However, in our work, the excess Te evaporates completely once the 1T′-MoTe_2_ layer has formed, This is because the excess Te will be evaporated during the laser irradiation process, yielding a conversion efficiency 100% of Mo to 1T′-MoTe_2_. Therefore, the local Te supply becomes insufficient to sustain the 1T′ → 2H transformation. Additionally, we fabricated a Mo/Te/Al_2_O_3_ sandwich on quartz to selectively produce the 2H-MoTe_2_ phase via the laser annealing process. A 10 nm-thick Mo layer was first deposited to maximize laser absorption, followed by 20 nm of Te as the chalcogen source and capped with 20 nm of Al_2_O_3_ to suppress Te volatilization during the laser annealing process. The quartz substrate does not absorb the 808 nm laser wavelength, ensuring that only the irradiated thin-film region undergoes solid-state reactions (Figure 2c, with the darker region outlined by the red dashed line). Additionally, the high density of Al_2_O_3_ effectively suppresses Te volatilization during the laser annealing process under atmospheric pressure. As a result, under a short laser dwell (6 W, 1 min), the converted regions exhibit phase-pure 1T′-MoTe_2_ confirmed by Raman results. Under prolonged irradiation (≥5 min at 6 W), 2H phase appears locally, yielding spatially mixed 1T′/2H domains rather than a uniform 2H phase film (Figure 2d). Note that the Raman spot (∼1 μm diameter) resolves >1 μm^2^ 2H patches, confirming that the 2H phase can be confined to a localized area with a higher thermal energy.^26^ In contrast, the superior thermal conductivity of Si substrates enables the fabrication of uniform 2 cm^2^ 1T′-MoTe_2_ films within 1 min, making them suitable for the subsequent back-gate FET fabrication. Thus, further material characterization will be conducted on 1T′-MoTe_2_ films synthesized on Si substrates. X-ray photoelectron spectroscopy (XPS) results, as shown in Figure 2e,f, reveal that after the laser annealing processes, Mo 3d_5/2_ and 3d_3/2_ peaks are located at 228.3 and 231.4 eV, respectively, while the Te 3d_5/2_ and 3d_3/2_ peaks appear at 572.9 and 583.1 eV. These binding energies are consistent with reported 1T′-MoTe_2_ values.^27^ Figure 2g presents a typical cross-sectional bright-field TEM image, showing a synthesized 1T′-MoTe_2_ film with a thickness of approximately ∼7.7 nm. The corresponding high-resolution transmission electron microscopy (HRTEM) further reveals its layered structure with a total thickness of 10–11 atomic layers along a stacking (010) plane, for which an internal space of 0.70 nm of 1T′-MoTe_2_ was indexed, as shown in Figure 2h.^28^ Additionally, scanning transmission electron microscopy (STEM) combined with energy-dispersive X-ray spectroscopy (EDX) confirms the Mo-to-Te atomic ratio of ∼1:2, verifying the stoichiometry of the synthesized material (Figure S2). Ultraviolet photoelectron spectroscopy (UPS) (Figure 2i) determines the work function of 1T′-MoTe_2_ to be approximately 4.82 eV, slightly lower than some theoretical values but within an acceptable range.^29^ To evaluate the feasibility of large-area fabrication, we prepared a 2 cm^2^ Mo/Te/Mo sandwich structure and performed the laser annealing process. The optical microscopy image (Figure 2j) shows that the Mo/Te/Mo film exhibits metallic clusters before the annealing process, whereas the annealed 1T′-MoTe_2_ film appears deep blue. The color change reflects alterations in refractive index and absorption properties because of the phase transformation. A Raman mapping image at a position of 520.6 cm^–1^ (Figure 2k) and Raman spectra at randomly nine measurement points (Figure 2l) confirm the uniform growth of the 1T′-MoTe_2_ film. Furthermore, we measured the sheet resistance of two 1T′-MoTe_2_ films synthesized under different laser powers (15 and 16 W), yielding values of 33 Ω/sq and 35 Ω/sq, respectively. These results indicate that the film quality is comparable under varying conditions, which is consistent with the Raman spectroscopy data and demonstrates the stability and reproducibility of the process. Additionally, the obtained sheet resistance values are in agreement with those reported in previous studies, further confirming the semimetallic nature of 1T′-MoTe_2_.^30^

**Figure 2:**
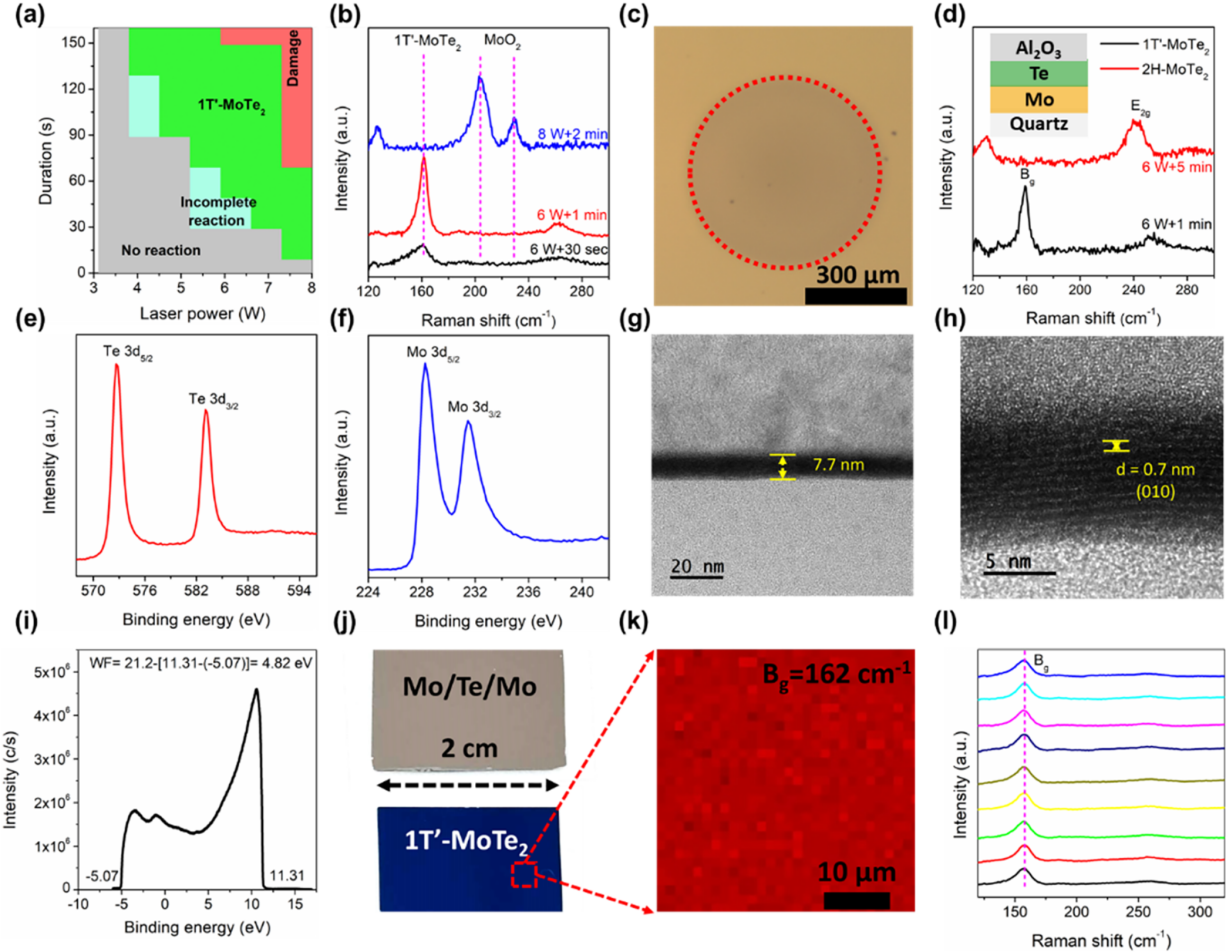
Synthesis and Material Characterization of MoTe_2_. (a) Parameter table illustrating laser power and irradiation time, with different colors representing the postannealing outcomes. (b) Raman spectra acquired under various laser conditions. (c) An optical microscopy (OM) image of the quartz substrate following the laser annealing. (d) Overlaid Raman spectra recorded at a fixed laser power of 6 W, with irradiation durations indicated by color (black: 1 min; red: 1 min). (e) XPS spectrum of the Te 3d region for 1T′-MoTe_2_. (f) XPS spectrum of the Mo 3d region for 1T′-MoTe_2_. (g, h) Cross-sectional TEM image of 1T′-MoTe_2_. (i) UPS spectra analysis and work function calculation of 1T′-MoTe_2_. (j) Photographs of the sandwich structure before annealing (silver appearance) and after annealing (deep blue appearance). (k) Raman mapping of the deep blue sample in (j). (l) Overlaid Raman spectra obtained from nine distinct regions of the sample in (j) following Raman analysis.

Given the semimetallic properties of 1T′-MoTe_2_ and its compatibility with the laser annealing processing, we plan to further investigate the fabrication and electrical characterization of 1T′-MoTe_2_-contact MoS_2_ back-gate FETs on Si substrates to evaluate its potential as a contact layer for electronic devices. To further validate using 1T′-MoTe_2_ as the source/drain contacts for 1L-MoS_2_ FET devices, we conducted DFT simulations via Vienna Ab Initio Simulation Package (VASP). Figure 3a (right) shows an optimized crystal structure of few-layer 1T′ MoTe_2_ on monolayer MoS_2_. From the electrostatic potential profile in Figure 3a (left), the barrier width (*w*_t_) and height (Φ_t_) were determined as 0.78 Å and 1.55 eV, respectively. Furthermore, it can be observed that there is minimal charge transfer from MoTe_2_ to MoS_2_, as indicated by the undistorted potential profile. From the Simmon model (at low bias: *qV* ≪ Φ_t_),^31,32^ the tunneling-specific resistivity (ρ_t_) can be calculated by $\rho_{t}={\left(\frac{{dJ}_{t}}{dV}\right)}^{-1}\approx \frac{4\pi^{2}\hbar w_{t}^{2}}{q^{2}}\frac{\exp\left(2\frac{{\left(2m_{e}\right)}^{1/2}}{\hbar }\alpha w_{t}\Phi_{t}^{1/2}\right)}{\frac{{\left(2m_{e}\right)}^{1/2}}{\hbar }\alpha w_{t}\Phi_{t}^{1/2}-1}$ . As a result, the tunneling resistivity (ρ_t_) induced by the tunneling barrier between the MoTe_2_ and MoS_2_ was calculated to be 5.31 × 10^–11^ Ω·cm^2^, significantly lower than other 2D spacers and/or dielectrics.^33–36^ The DOS plots in Figure 3b illustrate the mechanism behind the low contact resistance. The PLDOS of only MoS_2_ atoms within the heterostructure (upper panel, Figure 3b), reveals a dramatic shift of the Fermi level into the conduction band. This indicates a strong n-type doping effect and results in a degenerated MoS_2_ layer at the interface under a high concentration of free electrons (*n*_2D_ ≈ 2 × 10^13^ cm^–2^), leading to correspondingly low sheet resistance (*R*_SH,C_ ≈ 31.2 kΩ). This doping is induced by charge transfer from the semimetallic 1T′-MoTe_2_ (lower panel, Figure 3b). The resulting band alignment confirms that 1T′-MoTe_2_ and MoS_2_ form a near ideal contact with a negligible Schottky barrier. The Raman spectra in Figure 3c further confirm that MoS_2_ turns to degenerate under MoTe_2_ contacts. The A_1g_ Raman vibration mode of MoS_2_ with MoTe_2_ contacts is red-shifted at ∼3 cm^–1^, equivalent to (0.6–1.5) × 10^13^ cm^–2^ of electron density and 12–30 meV above the conduction band minimum. Furthermore, the experimental measurements were conducted to confirm our simulation results. Figure 3d illustrates the structure of a back-gated MoS_2_ FET device, where the contact layer is composed of 1T′-MoTe_2_ synthesized via the laser annealing process, and Pt serves as both the capping layer and the source/drain electrodes. The fabrication process is depicted in Figure S3. Notably, to mitigate the potential damage to MoS_2_ caused by high-energy electron beams during metal deposition, we first deposited a 10 nm Te layer, followed by 2 nm of Mo and a 40 nm Pt capping layer, and subsequently subjected the stack to the CW laser annealing process.^37^ Raman and photoluminescence (PL) results measured before and after the laser annealing process reveal that the characteristic peaks of MoS_2_ remain virtually unchanged, confirming that the MoS_2_ layer retains excellent structural stability throughout the laser annealing process (Figure S4). We further used depth-profiling X-ray photoelectron spectroscopy (XPS) to investigate the chemical bonding and elemental distribution within the Te/Mo/Pt stacking layer before and after the laser annealing process. For the as-deposited Te/Mo/Pt electrode, as shown in Figure S5a,b, the Te 3d_5/2_ and 3d_3/2_ peaks appeared at 573.3 and 584.0 eV after the Ar milling time of 4 min, respectively. As the Ar milling time increases, the Te 3d peak shifted approximately 0.5 eV toward lower binding energy, accompanied by a similar 0.5 eV leftward shift of the Mo 3d peaks. These shifts indicate that, during the deposition process, Mo and Te form stable Mo–Te bonds at the interface, likely facilitated by the high-energy electron beam used for Mo evaporation; this may also induce localized alloying at the interface.^38^ Depth-profiling XPS performed after the laser annealing process, as shown in Figure 3e,f, reveals that when the milling time reaches 4 min, Te 3d_5/2_ and Mo 3d_5/2_ peaks were observed at 572.7 and 228.2 eV, conforming the characteristic bonding of MoTe_2_. The subsequent stability of these peaks upon further milling process confirms the uniformity and completeness on the phase formation of MoTe_2_. Notably, an oxygen signal in regions adjacent to the SiO_2_ substrate, likely due to residual hydroxyl groups or oxygen incorporation during the deposition under suboptimal vacuum conditions (Figure S6). This oxygen may subsequently react with Mo during the laser annealing process to form MoO_2_, potentially increasing the contact resistance.^11^

**Figure 3:**
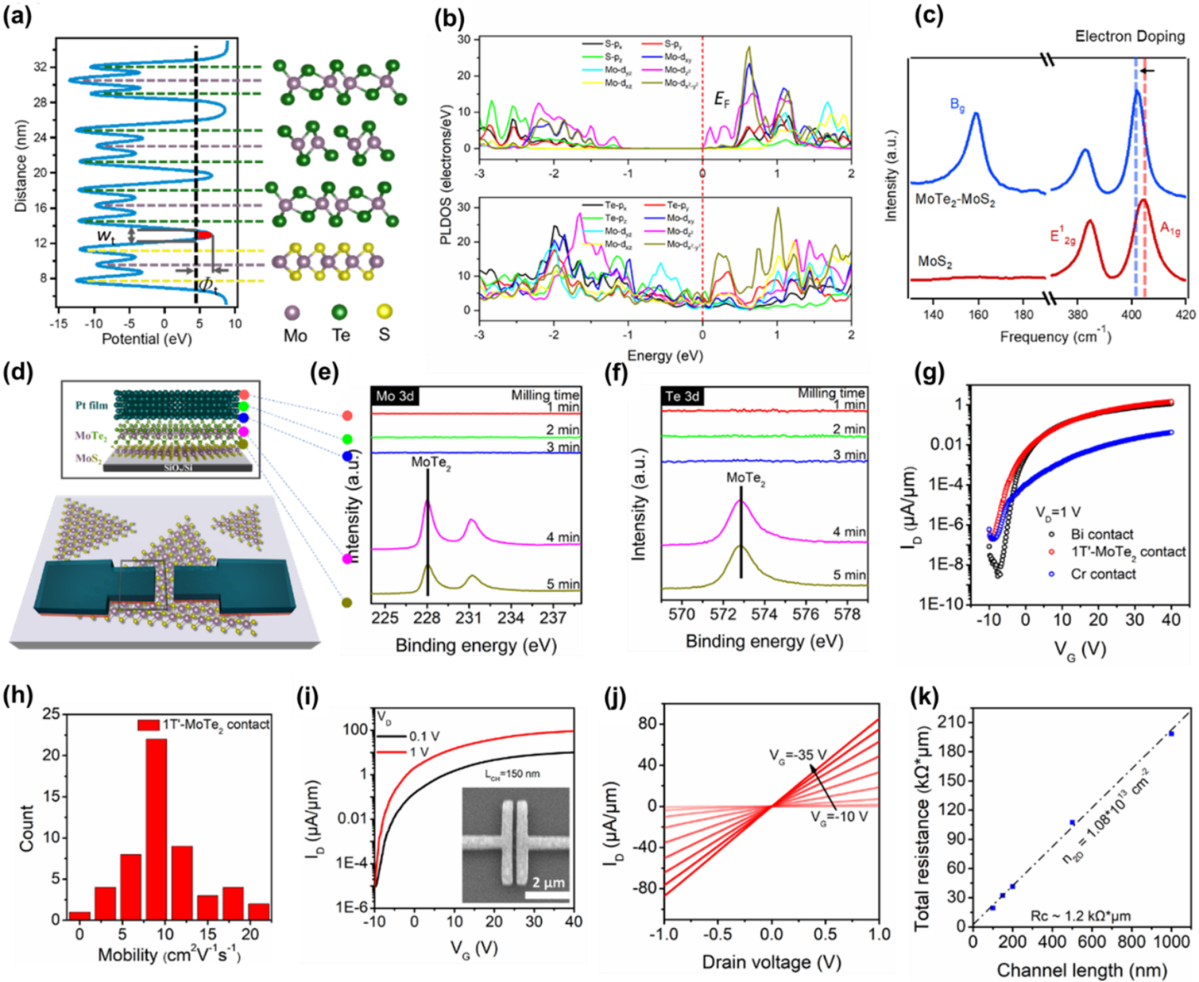
MoS_2_ FETs with 1T′-MoTe_2_ contacts Formed by the laser annealing. (a) Side-view of the optimized crystal structure and electrostatic potential profiles, (b) projected local density of states (PLDOS) for MoS_2_ (upper panel) after the contact contact with 1T′-MoTe_2_ (lower panel) and (c) Raman spectra of MoTe_2_–MoS_2_ heterostructure. (d) Schematic figures of a MoS_2_ FET with 1T′-MoTe_2_ contacts produced via the laser annealing. (e, f) XPS depth profiles for the electrodes, showing the (e) Mo 3d and (f) Te 3d spectra, respectively. (g) Overlaid transfer curves for MoS_2_ FETs with three different contact configurations. (h) Statistical distribution of the mobility for MoS_2_ FETs with 1T′-MoTe_2_ contacts. (i) transfer characteristics for a MoS_2_ FET with 1T′-MoTe_2_ contacts and a channel length of 150 nm; inset: SEM image of the device (scale bar: 2 μm). (j) Output characteristics of MoS_2_ FETs with 1T′-MoTe_2_ contacts. (k) Contact resistance extraction for the 1T′-MoTe_2_-contacted MoS_2_ devices using the TLM.

To reduce the additional interfacial resistance introduced by heterogeneous metal contacts, we initially attempted to use Mo alone as the electrode. However, following the laser annealing, devices with only Mo electrodes exhibited an on-state current density of only a few hundred nA/μm (Figure S7a). Further depth profiles using XPS (Figure S8) revealed that the unannealed Mo electrodes displayed significant oxygen signals at all depths, indicating oxidation during the annealing process and accounting for the elevated interfacial resistance. Nevertheless, as shown in Figure S7b, devices with an Au protective layer, after the laser annealing, exhibited an on-state current density of only tens of nA/μm. In Figure S9, atomic force microscopy (AFM) analysis of the post-annealed surface morphology revealed a *R*_a_ value of 16.3 nm. In contrast, devices annealed with a Pt protective layer exhibited a *R*_a_ value of approximately 0.43 nm under similar conditions. This discrepancy can be attributed to the dewetting phenomenon of the Au thin film at high temperatures during the laser annealing process,^39^ which compromises its role as an effective protective layer. Figure 3g presents the electrical performance of MoS_2_ FET devices that utilize 1T′-MoTe_2_ as the contact layer with Pt as a protective layer and source/drain electrodes. To compare different contact strategies, we fabricated devices with Au/Cr and Au/Bi as contact electrodes. We maintained a fixed channel length of 10 μm and calculated channel width from the average electrode coverage on the MoS_2_. The results show that devices with conventional Cr contacts achieve an on/off ratio of only 10^4^–10^5^, primarily due to the pronounced Fermi-level pinning effect, the formation of MIGS, and DIGS that arise during the deposition process. These factors collectively lead to higher Schottky barrier heights and increased contact resistance. In contrast, devices employing Bi and 1T′-MoTe_2_ contacts exhibit on/off ratios of 10^7^–10^8^. The superior performance of Bicontact FETs is attributed to its semimetallic nature and lower work function (approximately 4.1 eV), which mitigates the effects of MIGS. Moreover, 1T′-MoTe_2_ contacts, synthesized directly via the laser annealing process, demonstrate nearly equivalent electrical contact quality to Bi, yielding excellent device performance. Figure 3h summarizes the carrier mobilities with values ranging from 4 to 20 cm^2^/V·s and most devices clustering between 6 and 12 cm^2^/V·s, indicative of a highly uniform and stable contact interface between 1T′-MoTe_2_ and MoS_2_. To further highlight the advantages of the 1T′-MoTe_2_ contact layer, we fabricated devices with a channel length of only 150 nm, as shown in Figure 3i. These devices exhibit distinct transfer characteristics at drain voltage (*V*_D_) values of 0.1 and 1.0 V. At *V*_D_ = 1 V, the field-effect mobility was calculated to be approximately 10 cm^2^/V·s, the subthreshold swing (SS) is around 260 mV/dec, and the on/off ratio is about 7 orders of magnitude. To directly compare long- and short-channel behaviors, we plot transfer curves at *V*_D_ = 1 V for *L* ∼ 5 μm and *L* ∼ 150 nm devices (Figure S10). The short-channel device exhibits a smaller subthreshold slope (SS ∼ 260 mV/dec) and a lower threshold voltage (*V*_th_) ∼ 0 V than the long-channel device (SS ∼ 474 mV/dec; *V*_th_ ∼ 12 V), as extracted over a 2–3-decade linear window in the subthreshold region and by linear-extrapolation on the linear-scale *I*_D_–*V*_G_ plot, respectively. The corresponding transconductance efficiency *g*_m_/*I*_D_ = ln(10)/SS is ∼8.9 V^–1^ (short) versus ∼4.9 V^–1^ (long), evidencing stronger gate control in the short-channel device. We attribute this nontextbook trend to process-induced interface traps/fixed charges: the long-channel devices (optical lithography) likely retain a larger interfacial trap load than the e-beam-processed short-channel devices, consistent with the larger (*C*_it_ + *C*_d_)/*C*_ox_ inferred from SS (≈6.90 vs ≈3.33), where *C*_ox_ denotes the gate-oxide capacitance per unit area, *C*_it_ represents the interface trap capacitance, and *C*_d_ is the depletion capacitance associated with the underlying substrate. In addition, the transfer curves at *V*_D_ = 0.1 and 1.0 V show a noticeable drain-induced barrier-lowering-like shift, indicating that electrostatics can be further strengthened by gate-stack engineering. Although the device performance has not yet reached its optimum, these results suggest that further improvements are possible, for instance, by reducing the gate dielectric thickness to below a few nanometers or incorporating high-*k* materials, such as HfO_2_.^40^ Moreover, the output characteristics depicted in Figure 3j confirm the formation of a linear, low-Schottky ohmic contact between 1T′-MoTe_2_ and MoS_2_, indicating excellent interfacial quality. Using TLM at a carrier density of ∼1.08 × 10^13^ cm^–2^, devices with channel lengths ranging from 100 nm to 1 μm were measured, yielding a 1T′-MoTe_2_ contact resistance of ∼1200 Ω·μm (Figure 3k). The value is slightly higher than that observed for Bi semimetal contacts,^7^ possibly due to the presence of trace amounts of MoO_2_ at the interface, as observed in our previous XPS depth profiling analysis (Figure S6). Depth-resolved XPS of the as-deposited Mo film (Ar milling time = 0–4 min; Figure S11) shows that the oxidized Mo fraction, (Mo^4+^ + Mo^6+^)/Mo_total_, is the highest at the surface (∼38.5%) and drops to ∼ 6–12% at milling times of 1–3 min, consistent with a monotonic decrease of the O/Mo signal ratio with an increase in depth. The Mo^6+^/Mo^4+^ ratio likewise peaks at the surface (with Mo^6+^ ≈ 13% of total Mo) and becomes nearly negligible at longer milling time, indicating a surface-enriched high-valence oxide that transitions to a more substoichiometric subsurface oxide. Together, these trends indicate that interfacial MoO*_x_* is introduced during electron-beam evaporation—most plausibly from residual gases and target/chamber outgassing under elevated e-gun current—rather than being generated solely by the subsequent laser annealing process. Guided by this insight, suppressing oxygen uptake during Mo deposition should further reduce the contact resistance. Practical routes include: (i) tuning deposition parameters (lower e-gun current and deposition rate, extended pump-down and predegassing, higher-purity Mo targets), and (ii) improving base vacuum and monitoring (chamber bake-out, cryo/ion pumping, and residual-gas analysis). These refinements are expected to minimize interfacial MoO*_x_* formation and narrow the gap to state-of-the-art contacts.^11^

For BEOL processes, the interface between metal and the semiconductor channel is frequently exposed to various annealing conditions, posing a significant challenge to the long-term stability and performance of devices. To evaluate the thermal stability of different contact structures under the thermal processing, we annealed the devices in a horizontal furnace under a nitrogen atmosphere at temperatures ranging from 100 to 400 °C (with each step lasting 5 min). Subsequently, we measured the *I*_d_–*V*_g_ characteristics after each annealing cycle to analyze their transport properties. Figure 4a,b illustrate the electrical behaviors of FETs employing Pt/1T′-MoTe_2_ contacts and Au/Bi contacts under these varied annealing conditions. The Pt/1T′-MoTe_2_ contact layer was fabricated via the laser annealing of the Te/Mo/Pt layered structure. When the annealing temperature increases to 300 °C, as shown in Figure 4a, the *I*_d_–*V*_g_ characteristics of the device remain stable; even after annealing at 400 °C, the on/off ratio is maintained at approximately 7 orders of magnitude, demonstrating the excellent thermal stability of the 1T′-MoTe_2_ contact layer under high-temperature conditions. In contrast, although the on-state current density of MoS_2_–FETs with Au/Bi contacts increases slightly following the thermal annealing processes, the device characteristics almost completely degrade when the annealing temperature reaches 400 °C (Figure 4b). This result is consistent with previous reports,^41^ indicating that the thermal stability of Au/Bi contact layers is significantly inferior to that of Pt/1T′-MoTe_2_. In addition, we observed opposite shifts in the *V*_th_ following annealing for the two contact structures. Specifically, Pt/1T′-MoTe_2_ devices exhibited a positive *V*_th_ shift, which may be attributed to slight oxidation of the metal electrodes or oxygen adsorption on sulfur vacancies in MoS_2_. In contrast, devices using Au/Bi as the contact showed a negative *V*_th_ shift, likely due to the removal of processing contaminants and residuals during annealing.^42^ Notably, as shown in Figure S12, a negative *V*_th_ shift was observed in devices after the laser annealing, suggesting that the laser process effectively removed some of the impurities in the Pt/1T′-MoTe_2_ devices. Figure 4c,d present the on-state current (*I*_on_) and carrier mobility distributions for devices with Pt/1T′-MoTe_2_ contacts and Au/Bi contacts, respectively. The data show that devices with Pt/1T′-MoTe_2_ contacts maintain stable *I*_on_ and mobility across various annealing conditions, further confirming the protective role and excellent thermal stability of this contact layer on the MoS_2_ channel. In contrast, the *I*_on_ and mobility of Au/Bi devices progressively increase with the rising annealing temperature, resulting in complete device failure at 400 °C. To further explore the ultimate thermal endurance of the Pt/1T′-MoTe_2_ contact layer, we increased the annealing temperature to 500 °C. Figure S13 presents the *I*_d_–*V*_g_ measurements of short-channel devices subjected to thermal annealing in a nitrogen environment at temperatures ranging from 200 to 500 °C. Although device performance exhibits slight degradation after the thermal annealing at 400 °C (with previous reports indicating that significant Te evaporation from MoTe_2_ occurs at around 427 °C, leading to performance decline),^43^ the devices still retain discernible electrical behaviors after the thermal annealing at 500 °C. Complementary Raman spectroscopy before/after 500 °C annealing (Figure S14) confirms the persistence of 1T′-MoTe_2_ while revealing additional MoO_2_ peaks, indicating that the observed electrical degradation arises from interfacial oxidation. The result demonstrates that the Pt/1T′-MoTe_2_ contact layer remains functional under extremely high-temperature conditions, effectively simulating the stringent environment of the BEOL processing and providing robust experimental evidence for its application in high-temperature electronic devices. Moreover, considering the practical requirements of BEOL processes, these findings further confirm that this contact structure maintains stable electrical performance under extreme thermal stress. We further perform annealing tests of the Pt/1T′-MoTe_2_ contact at approximately 10^–1^ Torr, as shown in Figure S14. The on-state current is slightly enhanced and the SS is marginally reduced at an annealing temperature of 400 °C. This further substantiates that the Pt/1T′-MoTe_2_ contact exhibits stable and superior electrical performance under varying annealing conditions. These outcomes not only reinforce its potential as a thermally stable contact layer for high-temperature applications but also highlight its promise for next-generation, low-power, and high-reliability electronic devices.

**Figure 4:**
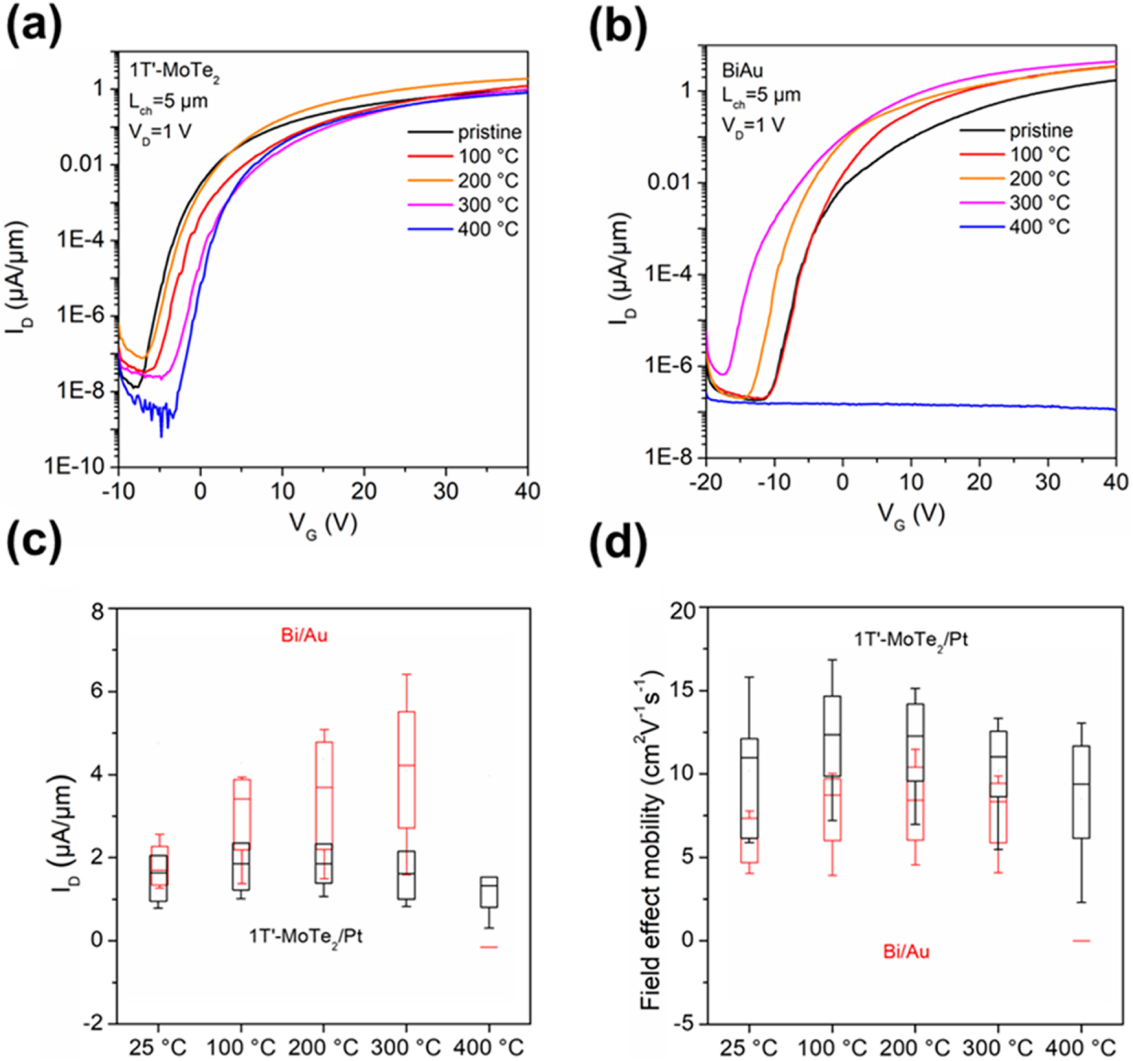
Transfer characteristics of FETs with different contact materials, followed by the furnace annealing for stability tests conducted at temperatures ranging from 100 to 400 °C. (a) Pt/1T′-MoTe_2_; (b) Au/Bi. (c) Statistical distribution of *I*_on_ for five devices measured at various annealing stages, where black corresponds to Pt/1T′-MoTe_2_ and red corresponds to Au/Bi. (d) Statistical distribution of mobility for five devices measured at various annealing stages, with black representing Pt/1T′-MoTe_2_ and red representing Au/Bi.

To evaluate the feasibility of our laser annealing process for large-area applications, we first fabricated a uniform MoS_2_ thin film on a 2 in. sapphire substrate (Figure 5a). Optical microscopy revealed that the MoS_2_ film exhibited uniform color and contrast across the entire substrate, indicating excellent consistency in both thickness and quality. Raman spectroscopy further confirmed the uniformity of MoS_2_ film, as the spectral features remained highly consistent across different regions, underscoring the repeatability and controllability of processes (Figure S16). Subsequently, we fabricated FET device arrays in a scale of 2 × 2 cm^2^ using the uniform MoS_2_ film (Figure 5b) and assessed the overall uniformity and layout precision via optical microscopy. An OM image clearly demonstrates that the spacing of device arrays, electrode coverage, and channel areas are highly uniform, reflecting the exceptional repeatability and precise control achieved in our large-area manufacturing process (An OM image at the right-hand side of Figure 5b). To assess the electrical uniformity of the device arrays, 50 devices were randomly selected, and their *I*_d_–*V*_g_ curves were overlaid (Figure 5c). The results indicate that these devices exhibit highly consistent *I*_on_, *V*_th_, and SS, confirming the excellent electrical uniformity of the arrays. Moreover, statistical analysis (Figure 5d1,d3) reveals that, compared to our previous small-area test results (Figure 3h), the SS, carrier mobility, and I_on_ of these devices display a significantly narrower distribution, indicative of high yield and reproducibility. Collectively, these results demonstrate that our laser annealing technique provides stable and scalable large-area processing, thereby underscoring its potential for advanced electronic device fabrication. Table S1 provides a side-by-side comparison with state-of-the-art transferred vdW contact methods. While our present contact resistance is higher than the best reported values for transferred vdW contacts, our approach offers a substantially simpler fabrication flow with a lower entry barrier and better compatibility with wafer-scale processing. Importantly, our depth-profiled XPS identifies oxygen uptake during Mo e-beam deposition as the dominant source of interfacial MoO_2_; we therefore expect that using higher-purity Mo targets and improved vacuum/pump-down protocols will further suppress oxidation and reduce contact resistance, narrowing the gap to the state-of-the-art.

**Figure 5:**
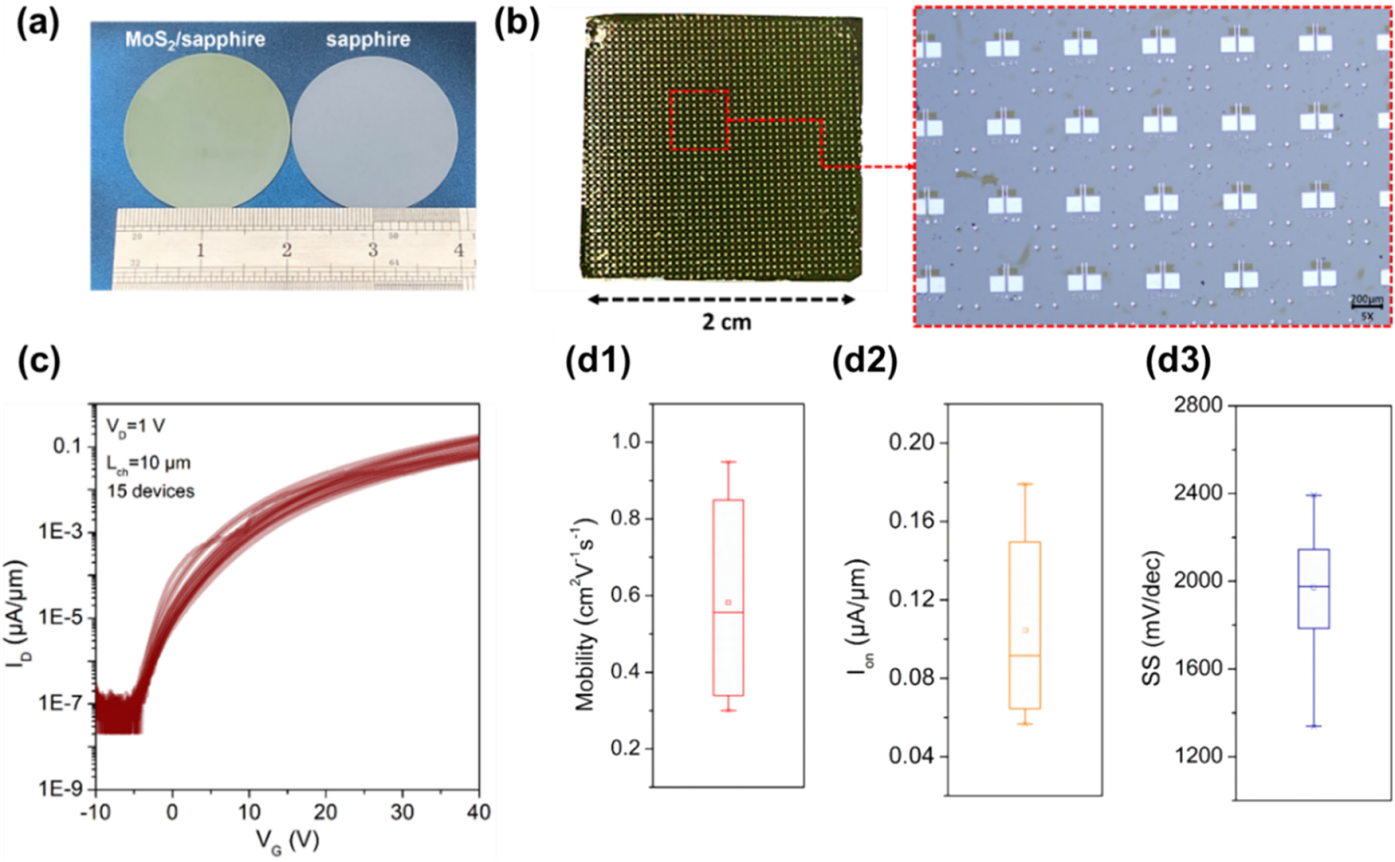
Laser-annealed large-area synthesized MoS_2_ FETs with 1T′-MoTe_2_ contacts. (a) 2 in. MoS_2_ film on sapphire. (b) An array photograph and optical microscopy image of MoS_2_ FETs with 1T′-MoTe_2_ contacts. (c) Transfer curves of 15 devices with a channel length of ∼10 μm, presented in logarithmic scale. (d1) Statistical distribution of mobility; (d2) Statistical distribution of *I*_on_; (d3) Statistical distribution of (SS).

## Conclusions

We present a laser-assisted, single-step synthesis of metal tellurides (MoTe_2_, Bi_2_Te_3_, PtTe_2_, and WTe_2_) that combines a low thermal budget, rapid kinetics, and uniform large-area coverage. Materials characterization confirms the formation of highly crystalline, layered 1T′-MoTe_2_. By tuning laser parameters and substrate selection, we achieve phase-selective growth of both 1T′ and 2H MoTe_2_. Direct CW laser synthesis of 1T′-MoTe_2_ on MoS_2_ yields FETs with field-effect mobilities of ∼10 cm^2^/V·s. Density functional theory reveals that gap-state saturation at the MoTe_2_–MoS_2_ interface effectively lowers contact resistance. While further device optimization remains possible, the 1T′-MoTe_2_ contacts retain an on/off ratio of 10^5^ after the thermal annealing at 500 °C, demonstrating both scalability and exceptional thermal stability. This work establishes a novel, practical route for integrating two-dimensional semimetal contacts into BEOL-compatible device architectures. Future investigations will focus on long-term thermal cycling and environmental exposure to fully qualify the reliability of these promising contacts for industrial applications.

## Methods

### CW Laser Annealing Process

Under high vacuum conditions (∼10^–6^ Torr), electron-beam evaporation was used to sequentially deposit the target metal (1–5 nm), tellurium (Te, 10–20 nm), and a metal capping layer (1–5 nm) onto a Si/300 nm SiO_2_ substrate. To ensure film uniformity, all deposition rates were maintained at 0.2 Å/s. The as-deposited samples were then transferred into a laser annealing chamber and evacuated to ∼10^–5^ Torr using a dry pump followed by a turbomolecular pump to prevent oxidation and contamination during annealing. Once the working pressure was reached, an 808 nm semiconductor laser was focused onto the sample via a lens assembly. The laser power was adjusted between 3 and 8 W for 5 mm^2^ samples, and annealing was performed for 1–5 min.

### Synthesis of Monolayer MoS_2_

Monolayer MoS_2_ single-crystal triangles were synthesized on sapphire substrates via atmospheric-pressure chemical vapor deposition (APCVD). Prior to the deposition, the sapphire substrates were sequentially ultrasonically cleaned in acetone, ethanol, isopropanol (IPA), and deionized water for 5 min each. After cleaning processes, the sapphire substrate and 2 mg of MoO_3_ powder (Alfa Aesar, 99.95% purity) were placed at the center of a quartz tube, and 1 mg of NaCl (Showa, 99.5% purity) was added as a catalyst to facilitate the reaction, while sulfur powder (Aldrich, 99.5–100.5% purity) was positioned in the upstream region. Initially, the 1 in. quartz tube was evacuated to 50 mTorr to ensure leak-free operation of the furnace. Subsequently, ultrahigh-purity argon (Ar) was introduced to raise the pressure to 760 Torr. The furnace was then heated to approximately 830 °C, while an alumina crucible containing sulfur powder was magnetically moved into the hot zone, enabling the sulfur to melt via the thermal gradient and promote the synthesis of MoS_2_. Following a growth period of 3 min, the CVD furnace was cooled to 500 °C, the furnace cover was opened to allow air cooling to 50 °C, and the sample was then carefully retrieved at this temperature.

### Transfer Method

The as-grown monolayer MoS_2_ film on the sapphire was spin-coated with PMMA (Kayaku, 950 PMMA A4) using a two-step process: first at 800 rpm for 10 s, followed by 2000 rpm for 30 s. After the spin-coating process, the film edge was gently scraped with tweezers to create a gap between the substrate and the PMMA/MoS_2_ stack, while minimizing surface damage. The separation was then achieved by immersing the PMMA/MoS_2_ stack in a diluted ammonia solution (NH_4_OH/DI water = 1:5). Subsequently, the separated PMMA/MoS_2_ stack was rinsed with deionized water and transferred onto a clean p^+^Si/50 nm SiO_2_ substrate. Finally, the transferred sample was heated at 70 °C for 20 min to remove residual moisture and enhance MoS_2_ adhesion, after which the PMMA layer was removed by soaking in acetone for 30 min. The sample was then further cleaned with isopropanol (IPA) and deionized water, and finally annealed in a vacuum furnace at 150 °C for 30 min in an Ar atmosphere to eliminate any remaining residues and contaminants.

### Fabrication of Back-Gate Transistors

Transferred MoS_2_ triangular flakes and films were utilized for the fabrication of back-gated FETs on p^+^Si/SiO_2_ (50 nm) substrates. The channel and source/drain regions were defined via photolithography using the photoresist AZ5241E. Electrodes were deposited by electron-beam evaporation at a deposition rate of <0.3 Å/s to ensure uniformity, under a pressure of approximately 10^–6^ Torr. The lift-off process was performed by immersing the device in PG stripping solution for 5–10 min to effectively remove the photoresist, followed by cleaning with IPA.

### Measurements and Characterization

Optical microscopy (OM) was employed to observe the morphology of the MoS_2_ samples. Raman spectroscopy (CL Technology, UniDRON) and photoluminescence (PL) measurements were conducted using a 532 nm laser at 50 mW, with the Si peak (520 cm^–1^) serving as the standard reference. Atomic force microscopy (AFM; Bruker, Dimension Icon) was used to analyze the surface roughness of the electrodes after laser processing. High-resolution transmission electron microscopy (HRTEM; JEOL, JEM-F200 operated at 200 kV) was performed at 200 keV to characterize the cross-sectional structure of the 1T′-MoTe_2_ synthesized via the laser annealing. XPS and UPS (UPS; Ulvac-PHI 1600) were utilized to measure the binding energies of the constituent elements and the work function of 1T′-MoTe_2_. Electrical measurements of the devices were carried out at room temperature under ambient conditions using a semiconductor parameter analyzer (Agilent, B1500A).

### First-Principles Calculations

The first-principles calculations, based on spin-polarized density functional theory (DFT),^44^ were performed using the Vienna Ab Initio Simulation Package (VASP)^45,46^ with the projector augmented plane wave (PAW) method.^47^ The exchange-correlation potential was treated within the generalized gradient approximation (GGA),^48^ specifically using the Perdew–Burke–Ernzerhof (PBE) framework.^49^ A kinetic energy cutoff of 500 eV was employed, with a self-consistent energy convergence criterion set to 10^–5^ eV. The structural relaxation was performed until the residual force on each atom was smaller than 10^–3^ eV/Å. In addition, the DFT-D2 Grimme method^50^ was included to incorporate the vdW correction during the relaxation. An out-of-plane vacuum of 10.0 Å was introduced to eliminate artifacts from periodic boundary conditions. A 5 × 3√3 supercell was used to construct the thin film structure, and a Γ-centered Monkhorst–Pack^51^ grid of 3 × 3 × 1 was used to model the first Brillouin zone.

## Supplementary Material



## Notes

Parameter table illustrating laser power and irradiation time (sample size: 1 cm^2^); STEM with elemental mapping; Schematic of fabrication processes for a back-gated MoS_2_ FET with Pt/1T′-MoTe_2_ contacts; Raman and PL spectra before and after the laser annealin; XPS depth profiles for electrodes before laser annealing process; Oxygen XPS depth profiles for the electrodes after the laser annealing process; Transfer curves of MoS_2_ FETs after the laser annealing process with Te/Mo and Te/Mo/Au contacts; Overlaid oxygen XPS depth profiles of Mo electrodes before the laser annealing process; AFM images of electrodes after the laser annealing process, with Pt and Au electrodes; Transfer characteristics of long-channel (5 μm) and short-channel (150 nm) devices, with the inset showing a magnified view of the selected region. Fitted Mo 3d spectra of the Mo film at different depths: surface, 10, 20, and 30 nm; Transfer curves of MoS_2_ FETs with Te/Mo/Pt contacts before and after the laser annealing process; Transfer characteristics of FETs with 1T′-MoTe_2_ contacts following furnace annealing for stability tests conducted at temperatures ranging from 200 to 500 °C (channel length: 500 nm); Ex situ Raman spectra acquired before (pristine, black) and after 500 °C annealing (red); Transfer characteristics of FETs with 1T′-MoTe_2_ contacts following furnace annealing for the stability tests conducted at temperatures from 300 to 400 °C under ∼10^–1^ Torr (channel length: 5 μm); Overlaid Raman spectra obtained from nine distinct regions of MoS_2_ film in 2 in. wafer; Comparison of transferred vdW contacts and this work (laser-direct 1T′-MoTe_2_) (PDF)
