# Supplementary material for: Direct Formation of Stable 1T′ Molybdenum Telluride (MoTe2) by Laser Annealing Processes as Robust Contacts for High-Performance Molybdenum Disulfide (MoS2) Field Effect Transistors
Source: ACS Appl Mater Interfaces. 2025 Oct 14;17(42):58520–31. doi: 10.1021/acsami.5c12868 (PMC12557218; doi:10.1021/acsami.5c12868)
Supplement: Supplementary file 1 [file am5c12868_si_001.pdf]

Supporting Information

Direct Formation of Stable 1T′ Molybdenum Telluride ($MoTe_2$) by Laser Annealing Processes as Robust Contacts for High-performance Molybdenum Disulfide (MoS_2) Field Effect Transistors

Yao-Zen Kuo[1,2,+], Ming-Jin Liu[1,2,+], Paul Albert Sino[1,2], Po-Chien Lai[1,2], Chia-Chen Chung[1,2], Yu-Chieh Hsu[1,2], Tzu-Yi Yang[1,2], Ruei-Hong Cyu[1,2], Feng-Chuan Chuang[3,4,5,6], Hao-Chung Kuo[7,8], Seokwoo Jeon[9], and Yu-Lun Chueh[1, 2, 3, 9*]

[1]Department of Materials Science and Engineering, National Tsing Hua University, Hsinchu 30013, Taiwan

[2]College of Semiconductor Research, National Tsing-Hua University, Hsinchu 30013, Taiwan

[3]Department of Physics, National Sun Yat-sen University, Kaohsiung 80424, Taiwan

[4]Physics Division, National Center for Theoretical Sciences, Hsinchu 30013, Taiwan

[5]Center for Theoretical and Computational Physics, National Sun Yat-sen University, Kaohsiung 80424, Taiwan

[6]Department of Physics, National Tsing Hua University, Hsinchu 30013, Taiwan

[7]Department of Photonics, College of Electrical and Computer Engineering, National Yang Ming Chiao Tung University, Hsinchu 30010, Taiwan

[8]Semiconductor Research Center, Hon Hai Research Institute, Taipei, 11492, Taiwan

[9]Department of Materials Science and Engineering, Korea University, Seoul, 02841, Republic of Korea

†These authors contributed equally to this work.

*E-mail address: ylchueh@mx.nthu.edu.tw

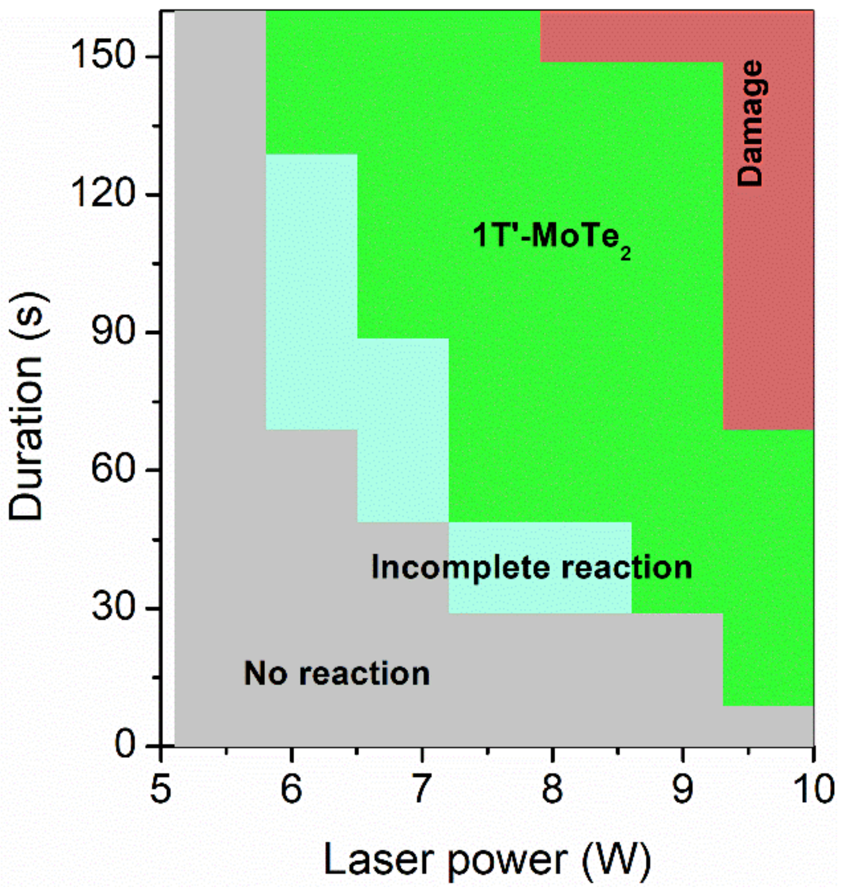

Figure S1 Parameter table illustrating laser power and irradiation time (sample size: 1 cm²).

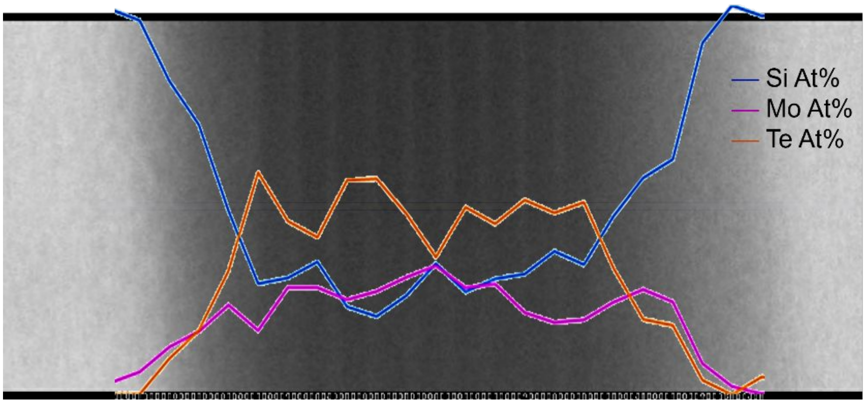

Figure S2 Scanning transmission electron microscopy (STEM) with elemental mapping of 1T'-$MoTe_2$.

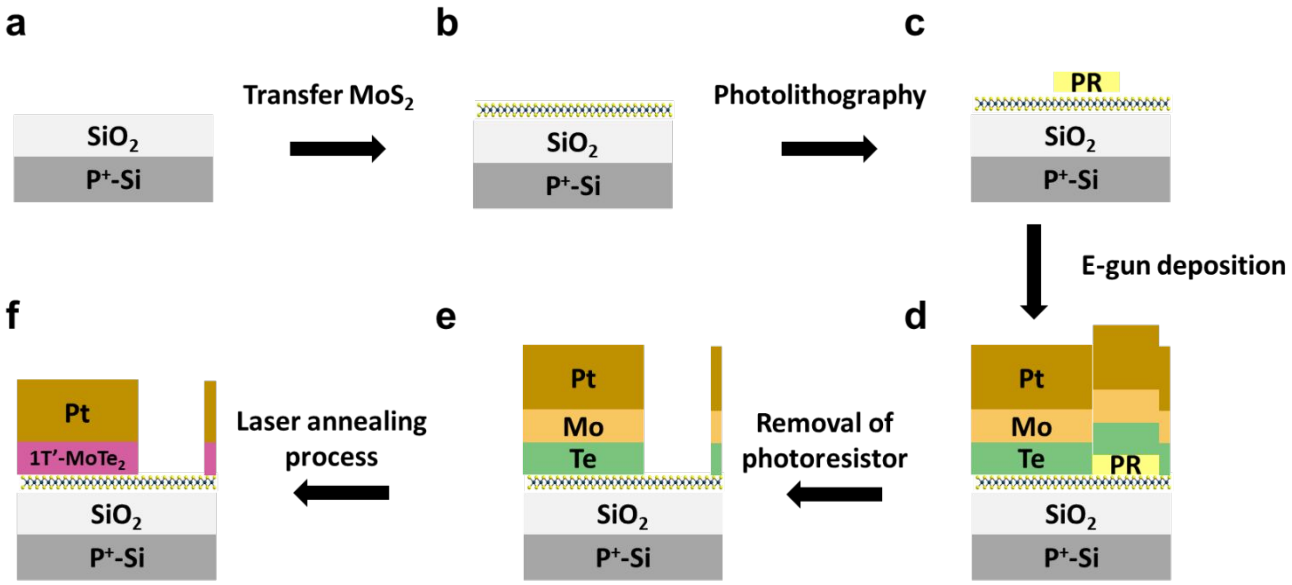

Figure S3 Schematics of fabrication processes for a back-gated MoS_2 FET using Pt/1T'-$MoTe_2$ contacts. (a) A pristine SiO_2/p$^+$-Si substrate. (b) Transfer of exfoliated MoS_2 onto the SiO_2/p$^+$-Si substrate. (c) Photolithography to define the source/drain regions. (d) E-beam deposition of Mo/Te/Pt layers with photoresist (PR) patterning. (e) Removal of the photoresist. (f) Formation of 1T'-$MoTe_2$/MoS_2 contacts after the laser annealing process.

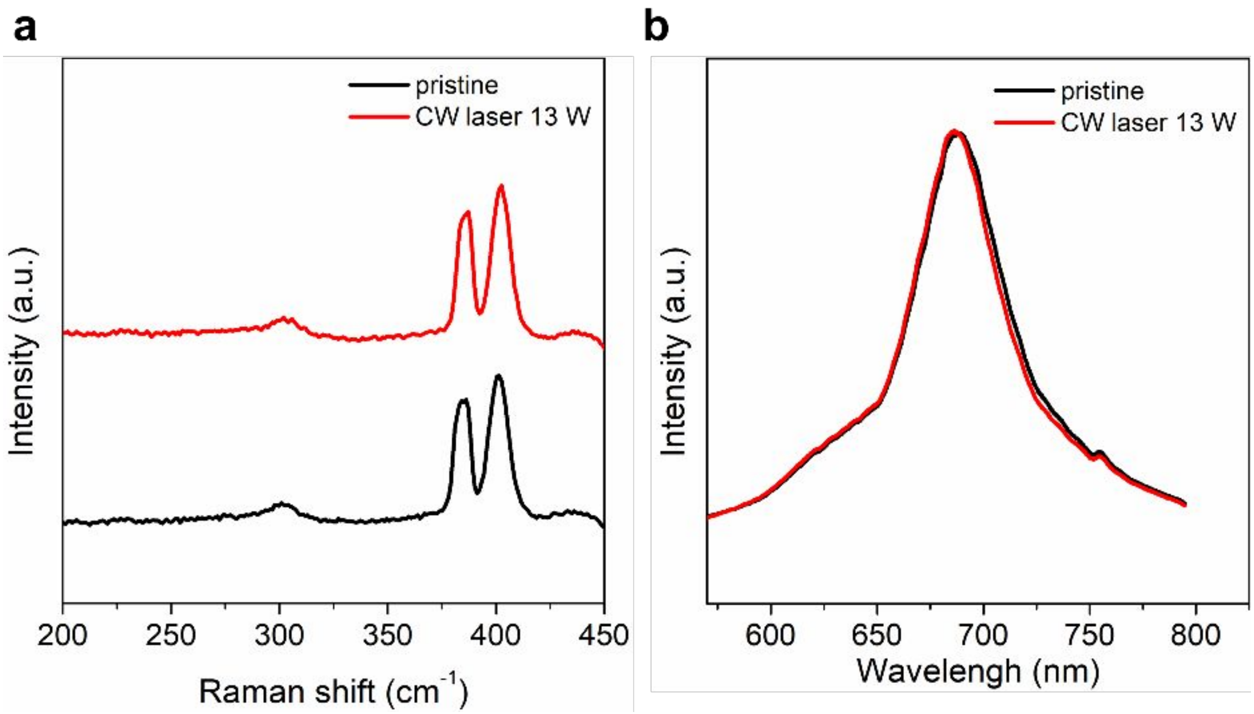

Figure S4 (a) Raman spectra before and after the laser annealing process; (b) PL spectra before and after the laser annealing process.

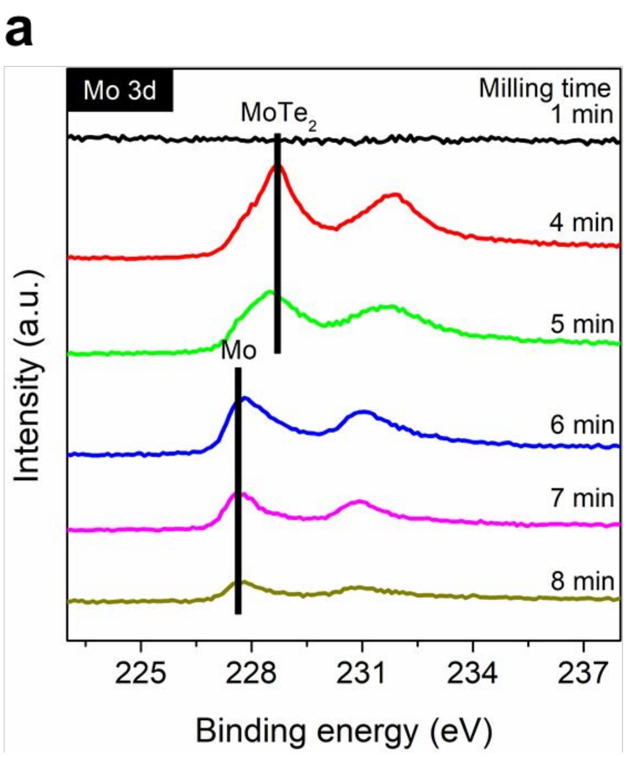

Figure S5 XPS depth profiles for the electrodes before the laser annealing process, showing (a) Mo 3d and (b) Te 3d spectra.

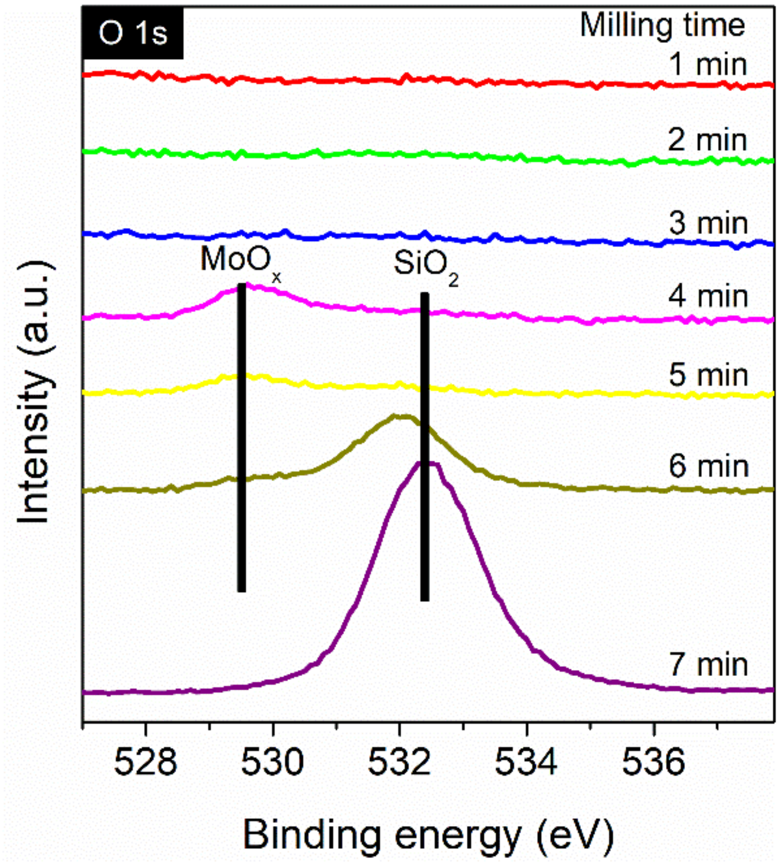

Figure S6 Oxygen XPS depth profiles for the electrodes after the laser annealing process.

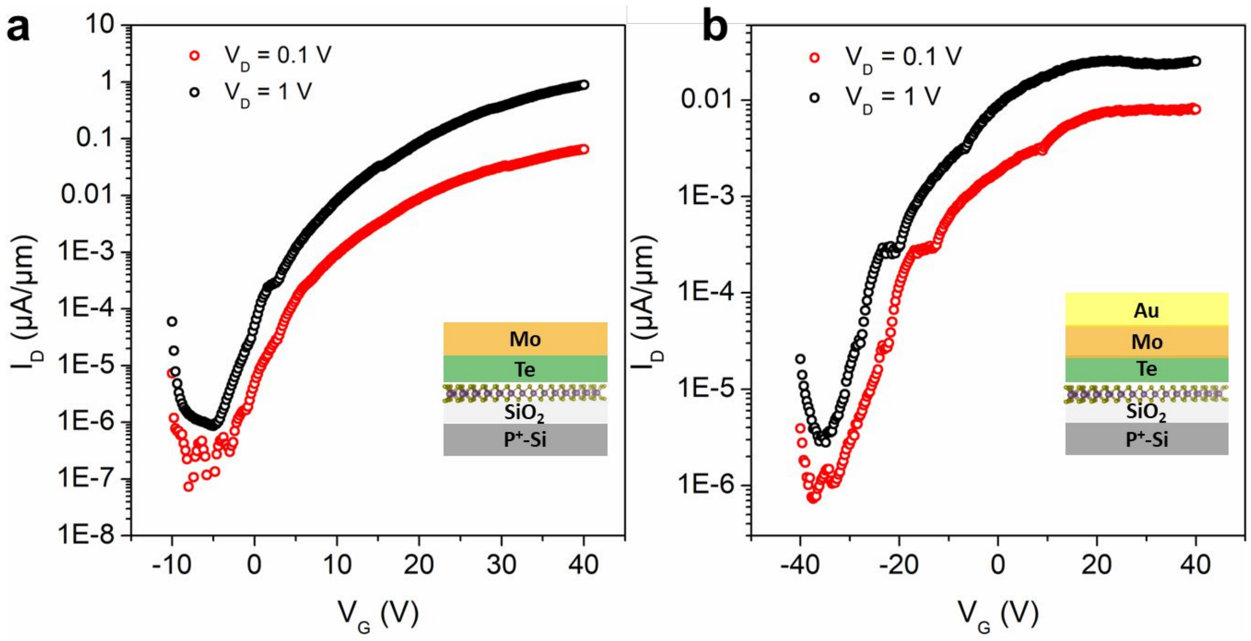

Figure S7 Transfer curves of MoS_2 FETs after laser annealing with (a) Te/Mo contacts and (b) Te/Mo/Au contacts.

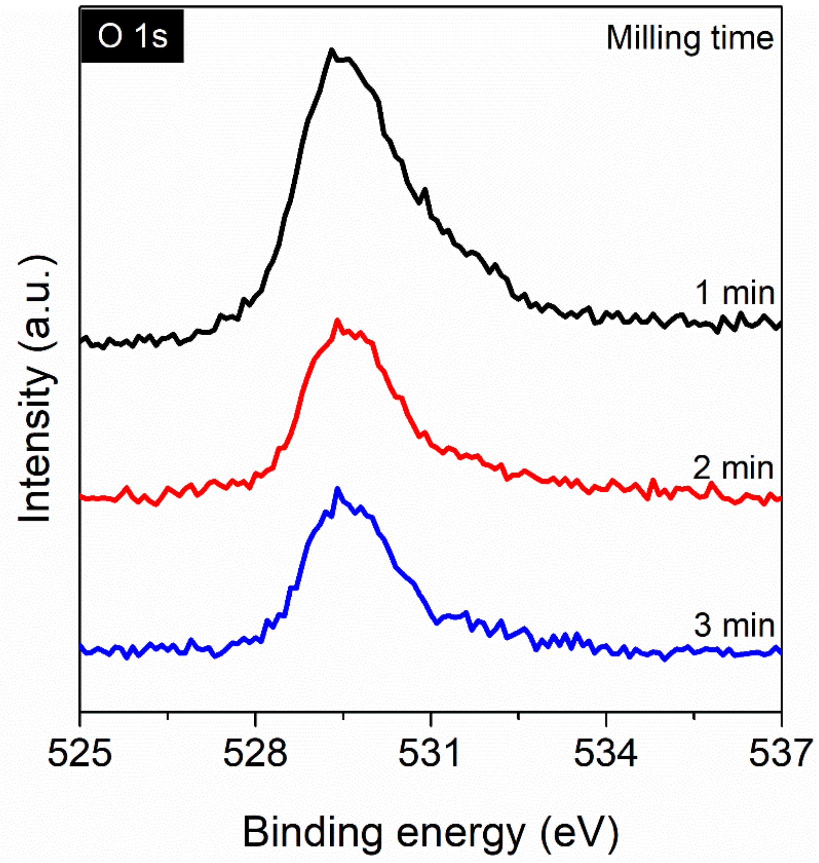

Figure S8 Overlaid oxygen XPS depth profiles of Mo electrodes before the laser annealing process.

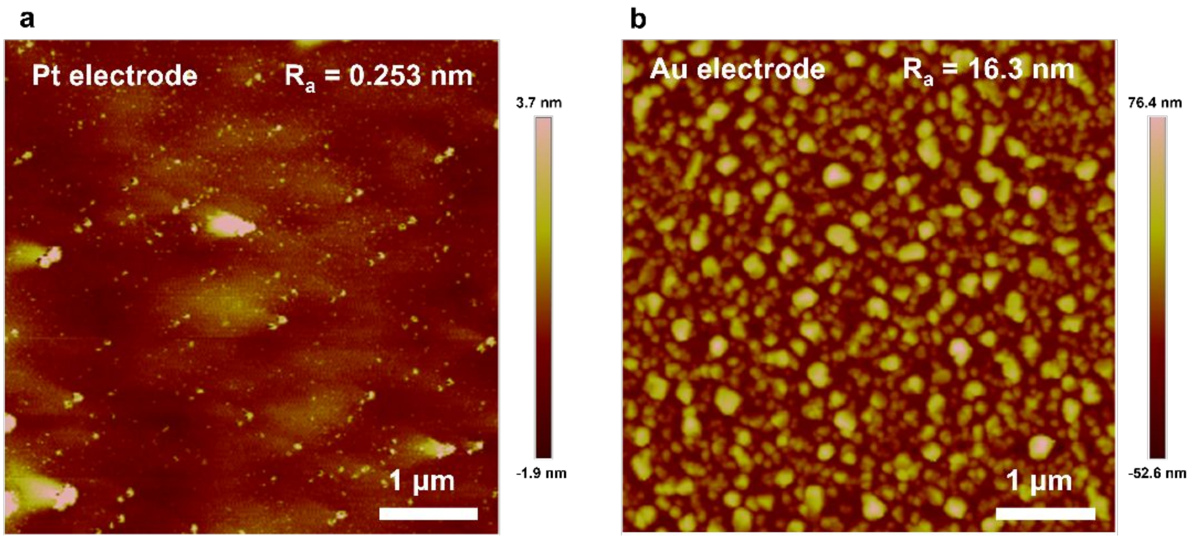

Figure S9 AFM images of electrodes after the laser annealing process, with (a) Pt electrodes and (b) Au electrodes, respectively.

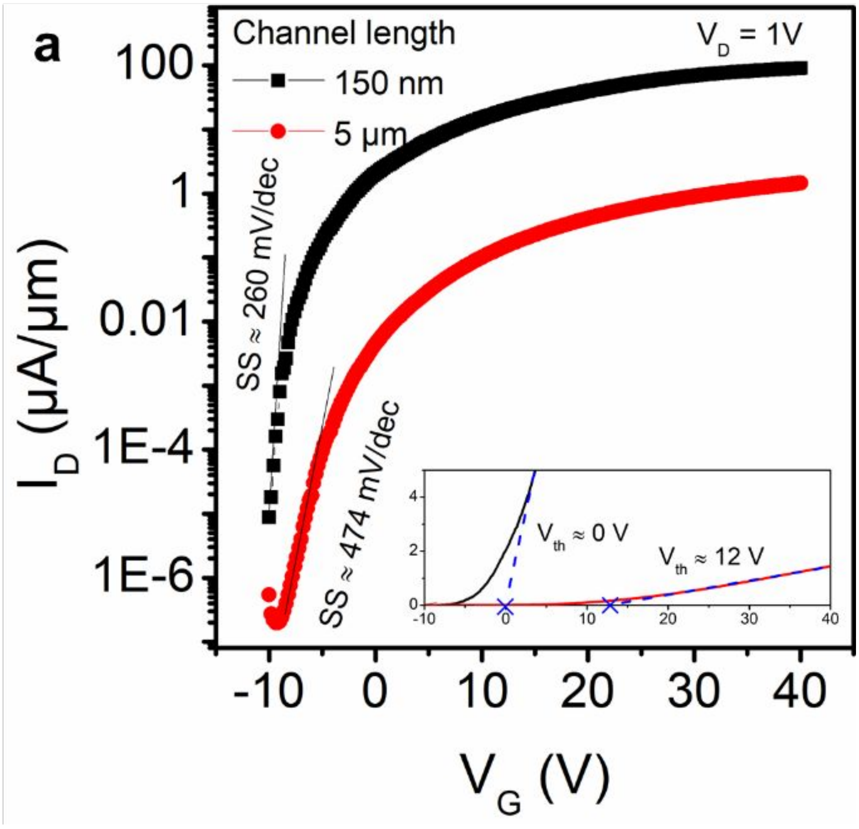

Figure S10 Transfer curves of long-channel (5 µm) and short-channel (150 nm) devices with the inset showing a magnified view of the selected region.

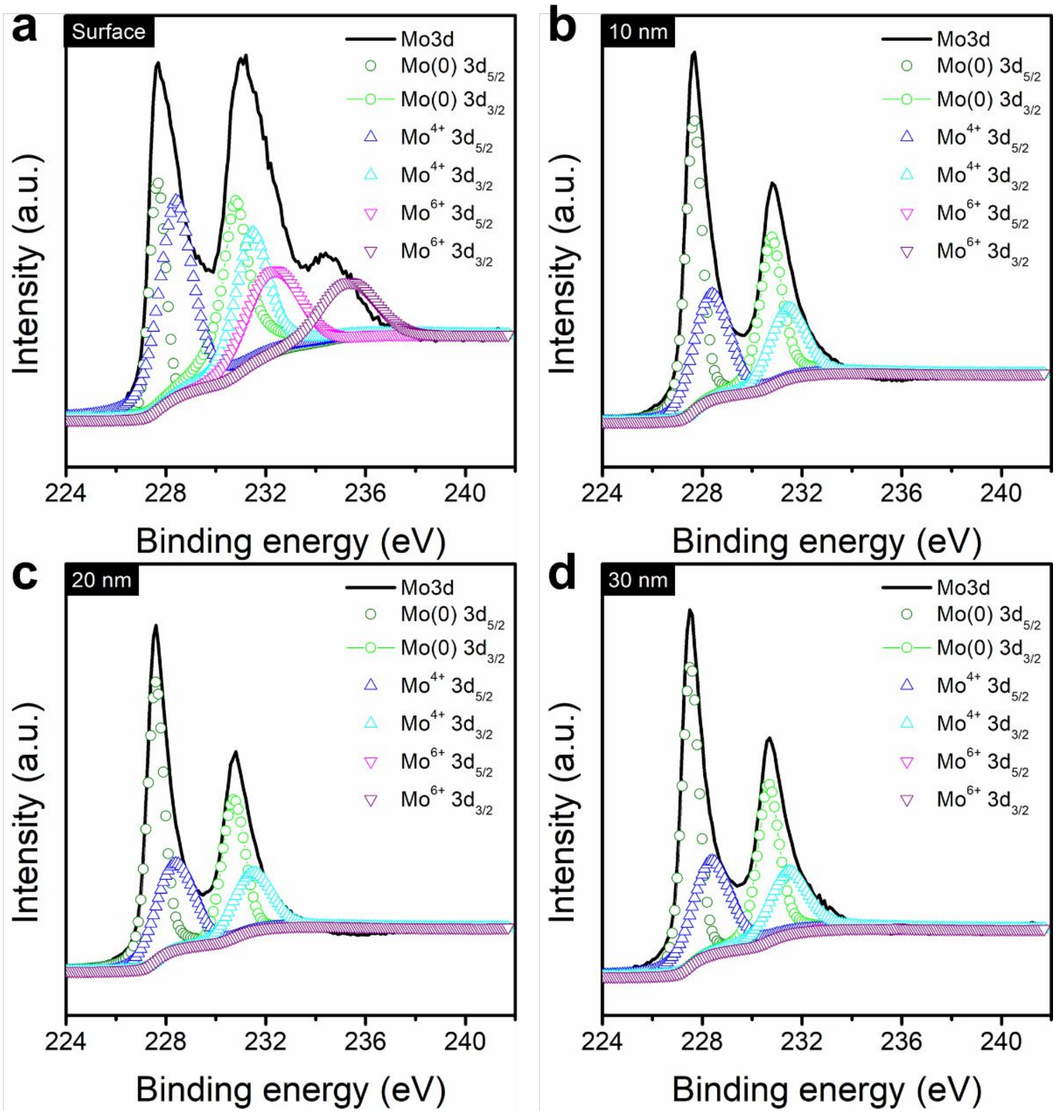

Figure S11 Fitted Mo 3d spectra of the Mo film at different depths: (a) surface, (b) 10 nm, (c) 20 nm, and (d) 30 nm.

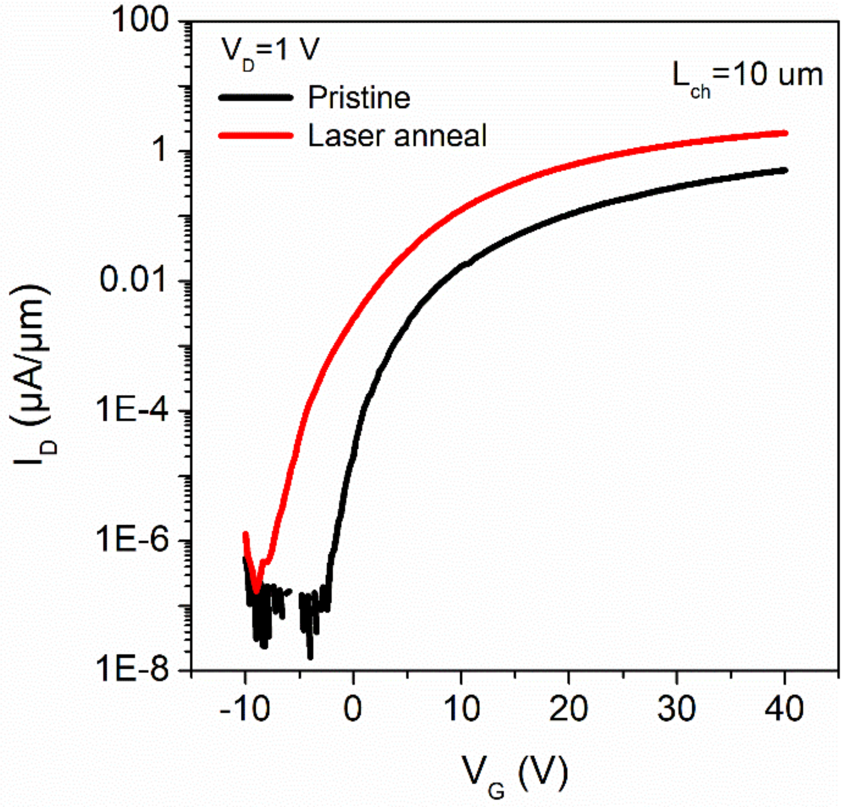

Figure S12 Transfer curves of MoS_2 FETs with Te/Mo/Pt contacts before and after the laser annealing process.

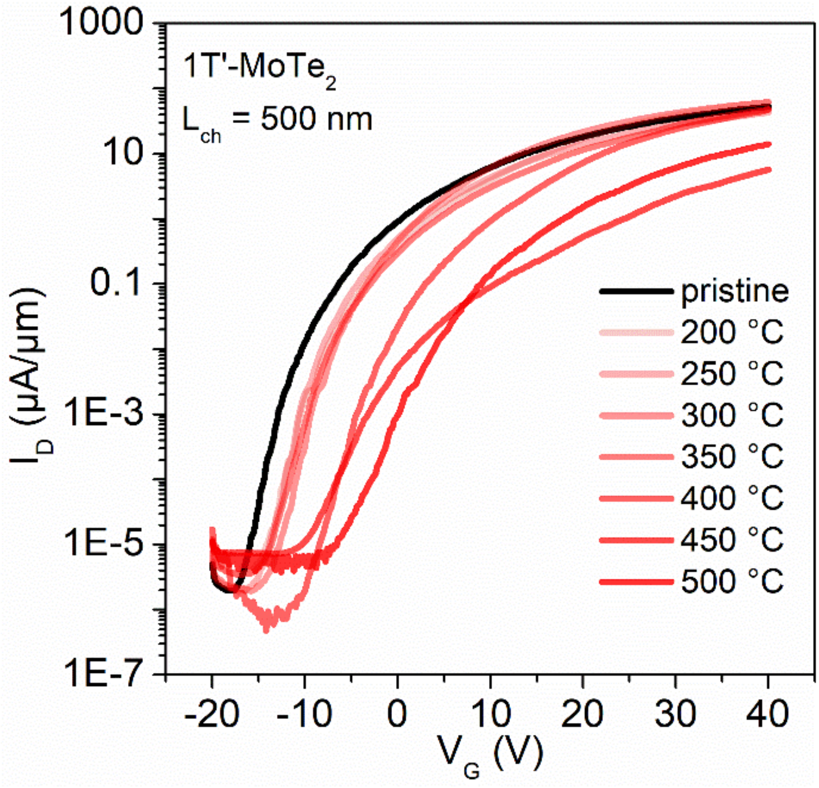

Figure S13 Transfer characteristics of FETs with 1T'-$MoTe_2$ contacts, followed by the furnace annealing process for stability tests conducted at temperatures ranging from 200 to 500 °C (channel length: 500 nm).

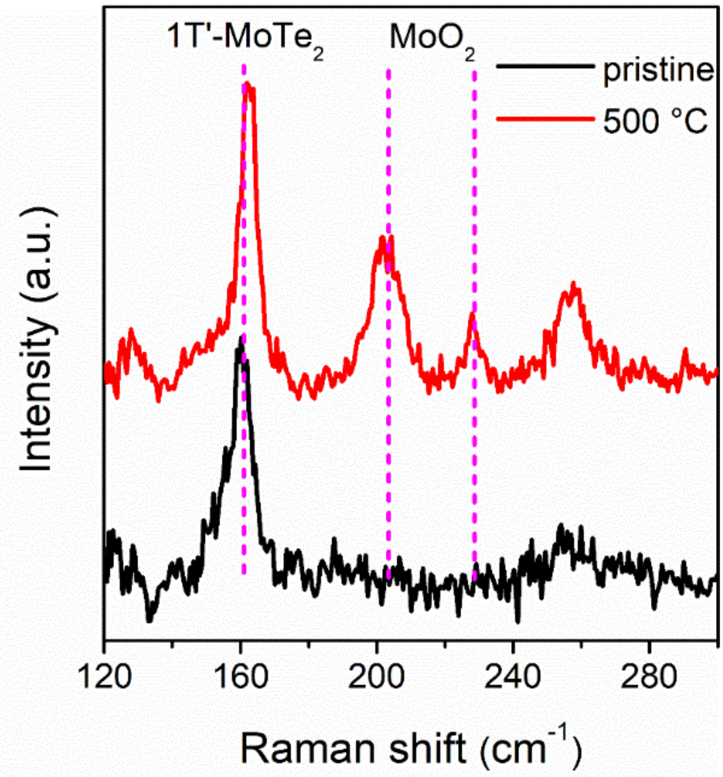

Figure S14. Ex-situ Raman spectra acquired before (pristine, black) and after 500 °C annealing (red).

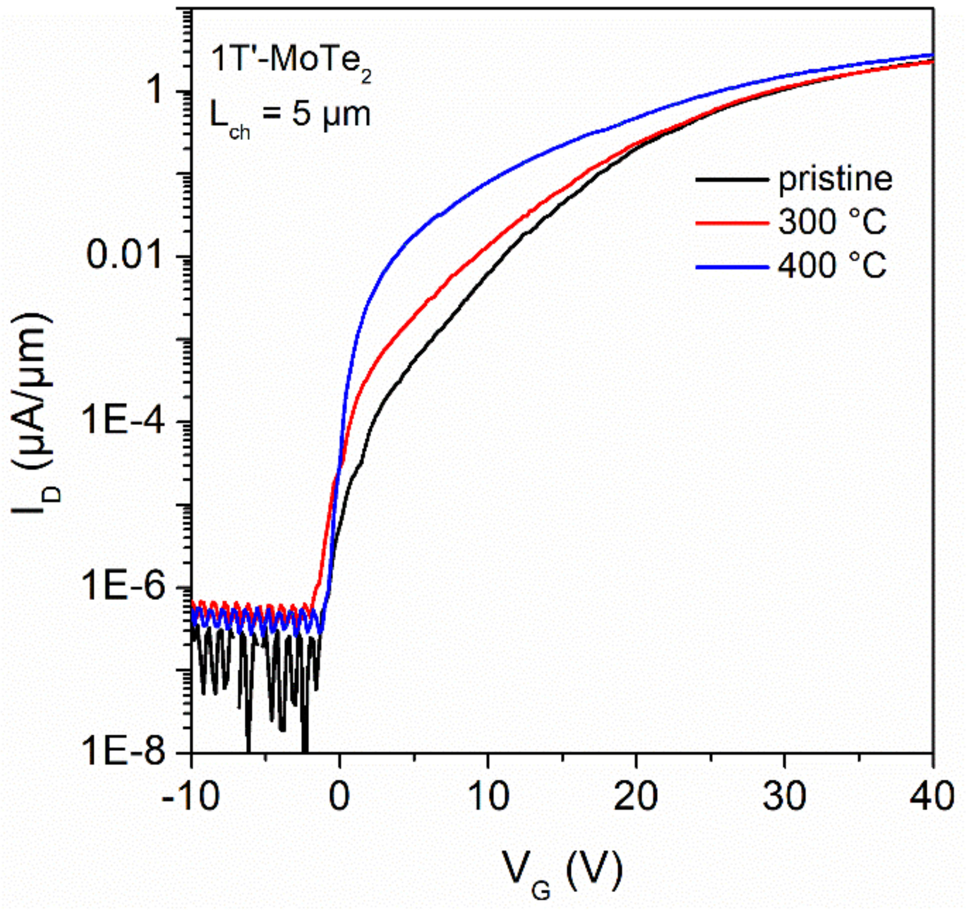

Figure S15 Transfer characteristics of FETs with 1T'-$MoTe_2$ contacts followed by the furnace annealing process for stability tests conducted at temperatures from 300 to 400 °C under ~10^{-1} Torr (channel length: 5 μm).

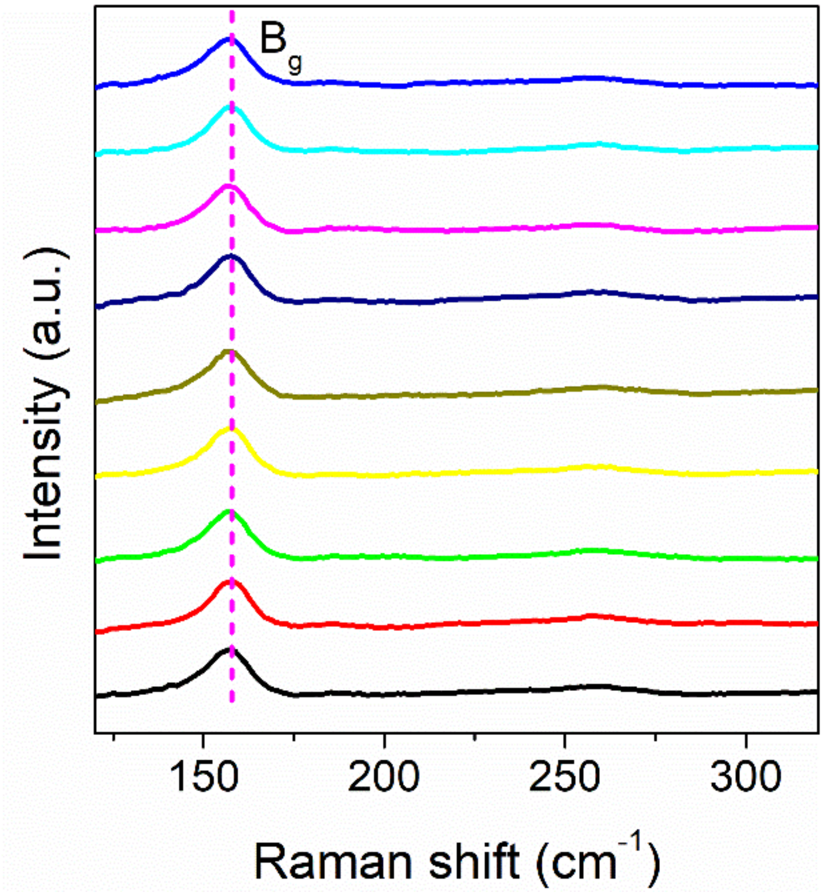

Figure S16 Overlaid Raman spectra obtained from nine distinct regions of MoS_2 film in 2-inch wafer.

Table S1. Comparison of transferred vdW contacts and this work (laser‑direct 1T'-$MoTe_2$)

Contact material (how obtained)	Rc (Ω·μm)	Large‑area demo?	BEOL (~400–450 °C) tolerance?	Process complexity	Ref.
Bi (semimetal) — thin film by thermal/ebeam evaporation	≈78 @ 15 K	Yes (cm‑scale platform)	No	Medium	[1]
ZrTe2 (Dirac semimetal) — exfoliated single‑crystal flake	≈42	Device‑scale (not wafer)	Yes —up to 450 °C, with h-BN encapsulation	High	[2]
PtTe2 (Dirac semimetal) — tellurization of patterned Pt thin films	≈1580	Device/array‑scale	N/R (no explicit BEOL‑temperature test in device context)	High	[3]
TiS2 (2D metal) — exfoliated flake	≈7755	Device‑scale	N/R (no explicit BEOL‑temperature test found)	High	[4]
Au&Al One‑Step electrode transfer — pre‑patterned metal electrodes prepared ex‑situ;	≈400	Yes (wafer‑scale aligned transfer)	N/R	Medium–High	[5]
This work: 1T′‑MoTe2 — laser‑direct formation on Mo/Te stack (transfer‑free, mask/laser defined)	≈1200	Yes (mask/laser‑defined, patternable)	Yes —up to 500 °C	Low	This work

Note: RT = room temperature; N/R = not reported. Complexity scale (qualitative): low = transfer‑free or single‑step patterning; medium = one transfer + alignment/standard release‑clean; high = growth of 2D metal + transfer/alignment and/or multiple transfer/clean steps.

References:

(1) Mondal, A.; Biswas, C.; Park, S.; Cha, W.; Kang, S.-H.; Yoon, M.; Choi, S. H.; Kim, K. K.; Lee, Y. H. Low Ohmic contact resistance and high on/off ratio in transition metal dichalcogenides field-effect transistors via residue-free transfer. *Nature Nanotechnology* **2024**, *19* (1), 34-43. DOI: 10.1038/s41565-023-01497-x.

(2) Wen, X.; Lei, W.; Li, X.; Di, B.; Zhou, Y.; Zhang, J.; Zhang, Y.; Li, L.; Chang, H.; Zhang, W. ZrTe2 Compound Dirac Semimetal Contacts for High-Performance MoS2 Transistors. *Nano Letters* **2023**, *23* (18), 8419-8425. DOI: 10.1021/acs.nanolett.3c01554.

(3) Ren, J.; Wang, L.; Yao, Q.; Zhang, L.; Dong, G.; Gao, Y.; Li, X.; Yang, C.; Li, Z.; Deng, K.; et al. Highly Efficient Electrode of Dirac Semimetal PtTe2 for MoS2-Based Field Effect Transistors. *ACS Appl. Mater. Interfaces* **2025**, *17* (1), 1469-1476. DOI: 10.1021/acsami.4c15095.

(4) Yoon, H.; Lee, S.; Seo, J.; Sohn, I.; Jun, S.; Hong, S.; Im, S.; Nam, Y.; Kim, H.-J.; Lee, Y.; et al. Investigation on Contact Properties of 2D van der Waals Semimetallic 1T-TiS2/MoS2 Heterojunctions. *ACS Appl. Mater. Interfaces* **2024**, *16* (9), 12095-12105. DOI: 10.1021/acsami.3c18982.

(5) Liu, J.; Xing, K.; Li, L.; Zhao, W.; Stacey, A.; Robertson, I.; Broadway, D. A.; Tetienne, J.-P.; Qi, D.-C.; Fuhrer, M. S.; et al. One-Step Transfer of Symmetric and Asymmetric Contacts for Large-Scale 2D Electronics and Optoelectronics. *ACS Nano* **2025**, *19* (30), 27919-27929. DOI: 10.1021/acsnano.5c09815.
